# Valuations of target items are drawn towards unavailable decoy items due to prior expectations

**DOI:** 10.1093/pnasnexus/pgae232

**Published:** 2024-06-24

**Authors:** Liz Izakson, Minhee Yoo, Adam Hakim, Ian Krajbich, Ryan Webb, Dino J Levy

**Affiliations:** Sagol School of Neuroscience, Tel Aviv University, P.O. Box 39040, Tel Aviv 6997801, Israel; Department of Psychology, The Ohio State University, 1835 Neil Avenue, Columbus, OH 43210, USA; Sagol School of Neuroscience, Tel Aviv University, P.O. Box 39040, Tel Aviv 6997801, Israel; Department of Psychology, University of California Los Angeles, 1285 Franz Hall, Box 951563, Los Angeles, CA 90095, USA; Rotman School of Management, University of Toronto, 105 St George St., Toronto, Ontario, M5S 3E6, Canada; Sagol School of Neuroscience, Tel Aviv University, P.O. Box 39040, Tel Aviv 6997801, Israel; Coller School of Management, Tel Aviv University, P.O. Box 39040, Tel Aviv 6997801, Israel

**Keywords:** decision making, context, assimilation, sequential sampling model, drift-diffusion model

## Abstract

When people make choices, the items they consider are often embedded in a context (of other items). How this context affects the valuation of the specific item is an important question. High-value context might make items appear less attractive because of contrast—the tendency to normalize perception of an object relative to its background—or more attractive because of assimilation—the tendency to group objects together. Alternatively, a high-value context might increase prior expectations about the item's value. Here, we investigated these possibilities. We examined how unavailable context items affect choices between two target items, as well as the willingness-to-pay for single targets. Participants viewed sets of three items for several seconds before the target(s) were highlighted. In both tasks, we found a significant assimilation-like effect where participants were more likely to choose or place a higher value on a target when it was surrounded by higher-value context. However, these context effects were only significant for participants’ fastest choices. Using variants of a drift-diffusion model, we established that the unavailable context shifted participants’ prior expectations towards the average values of the sets but had an inconclusive effect on their evaluations of the targets during the decision (i.e. drift rates). In summary, we find that people use context to inform their initial valuations. This can improve efficiency by allowing people to get a head start on their decision. However, it also means that the valuation of an item can change depending on the context.

**Significance Statement**

Our research studies how the context in which items are presented influences people's valuations and decisions. We found that the initial presence of surrounding items affects valuations and choices. When valuable items are nearby, target items tend to be perceived as more attractive and valuable. This effect was most pronounced when participants made quick decisions. By employing computational modeling, we discovered that context primarily shifted participants’ initial expectations, attracting them to the overall value of the items in the set. This can improve efficiency by allowing people to get a head start on their decision. However, it also means that surrounding context can be manipulated to change the valuation of an object, with implications for both marketing and consumer welfare.

## Introduction

We live in a busy world, surrounded by multitudes of stimuli that are processed by limited brains. Due to this limitation, we perceive, evaluate, and decide in a manner that allows us to conserve cognitive resources, but which also creates dependencies and relationships between stimuli and prior information (1). The question we ask here is how this bears on consumer choice. We consider two possibilities.

### Biases of product evaluations due to prior expectations

One possibility is that our brains use contextual information to construct prior expectations about the value of alternatives. Decisions take time, but time is valuable. To help speed up the choice process, a decision-maker can use prior information to inform their decisions. For example, a Los Angeles weather forecaster need not assume that rainy and sunny days are equally likely. By beginning their evaluation closer to the “sunny” conclusion, they can save time on the many sunny days at the cost of being slower on the few rainy days, while slightly overpredicting the number of sunny days. This is a case of using base-rate information (i.e. temporal context) to inform choice and save time.

Another source of information that people may use to speed up their decisions is the surrounding context (i.e. spatial context). For instance, shoppers may use the environment they are in to inform their purchase decisions for individual items. Consider a shopper debating whether to purchase a chocolate bar. If they are visiting Belgium, the shopper may be faster, more likely to purchase, or be willing to pay more for the same candy bar than if they were visiting Iceland, because of their prior expectation that Belgium has great chocolate, while Iceland does not. Because consumer evaluations are noisy and imprecise (like weather forecasting), people may use context to inform and speed up their decisions, even at the cost of misvaluing an item.

### Biases of product values due to relative evaluation

Another possibility is that context may also affect the way that items are perceived or evaluated. Efficient perception creates dependencies and relationships between stimuli (2, 3). These principles are evident both in the sensory domain, through perceptual heuristics and illusions (4), and in the value domain, through relative valuation processes that explicitly depend on context (5–10).

Contrast effects occur when the judgment of a target stimulus is biased away from the context (11). For example, the Ebbinghaus illusion is a visual perceptual phenomenon where the perceived size of a central target object is affected by the size of the objects surrounding it: a target circle tends to be perceived as smaller when it is surrounded by larger circles and larger when it is surrounded by smaller circles (12). In the value-based domain, the relative probability of choosing the highest-value product from a choice set is lower in the presence of a higher-value distractor compared to a lower-value distractor (13, 14) (but see also (15) and replies by Webb et al. (16) and Gluth et al. (17)), and faces are judged as more attractive when they are presented alongside less attractive faces (18). One possible explanation for this effect is Divisive Normalization, a canonical neural computation that is also thought to underlie the influence of context on perception across a range of sensory modalities (19–22).

In comparison, assimilation effects occur when the judgment of a target stimulus is biased towards the context (11). In the Eriksen flanker task, a target stimulus is more easily identified when it is surrounded by congruent flankers compared to incongruent ones (23, 24). When white and black striations are superimposed onto the same gray background, the intervening gray areas are judged lighter for the white stripes and darker for the black stripes (25, 26). A pin-cushion that is formed by four arcs on a gray background appears darker when the arcs are black, and lighter when the arcs are white (27). In economic choice environments, price and perceived quality of products are positively correlated (28), and store reputation positively affects product judgments (29) and customer purchase intentions (30). Moreover, people perceive unfamiliar products as more attractive and higher quality when they are placed in a more attractive context (31).

### Establishing the effects of context in consumer choice

In this article, we examine the effect of context on incentivized consumer choice. Specifically, we examine to what extent context affects values via prior expectations, relative evaluations, or both. We asked participants to provide their willingness-to-pay (WTP) or to choose between consumer products embedded in larger sets of products. Our design is analogous to how context is often manipulated in perceptual tasks (32, 33). Specifically, we used a version of the “naïve forced-exposure” (aka phantom decoy) design in which the context products are explicitly excluded from the choice set and cannot be chosen. The advantage of this design is that it includes both high- and low-value products serving as contexts relative to the target, a feature otherwise not possible when high-value context items can be chosen. Therefore, we can systematically examine the effect of both high and low contexts on target products. Additionally, we conducted both binary-choice and WTP experiments to test whether the effect of spatial context directly alters the perceived value of alternatives. The WTP experiment is important because it addresses the possibility that context alters final valuations rather than simply creating a response bias.

We then use computational modeling to examine the two mechanisms through which context can affect choice. We can identify the effect of context on both prior expectations and evaluations using the drift-diffusion model (DDM) (34). In the DDM, the influences of context before and during the decision/valuation process are captured by the starting point and drift rate (i.e. evidence accumulation rate), respectively. The starting point and drift rate are two distinct mechanisms that are affected by different experimental manipulations, and have different effects on the joint distributions of choice and response time (RT) (35–41). Starting points primarily affect the fastest responses, while drift rates primarily affect slower responses. Starting points provide a head start to one option over the other, narrowing the gap to one of the choice boundaries, while drift rates determine how quickly the evidence for the options approach those boundaries. As in any race, head starts are most impactful for short contests, while the relative speeds of the racers are most impactful for long contests. Thus, by analyzing the link between choice and RT, we can identify whether the context effects are likely due to prior expectations or evaluations (Table 1).

**Table 1.** Context effects.

		RT quintiles				
		1 (fast)	2	3	4	5 (slow)
Experiment 1 (Choice)	High target	0.04*** (<0.001)	0.02*** (0.003)	0.01 (0.07)	0.01 (0.42)	−0.00003 (0.996)
	Low target	−0.05*** (<0.001)	−0.02** (0.01)	−0.01* (0.05)	−0.01 (0.15)	−0.01 (0.38)
Experiment 2 (WTP)	Online	0.08*** (<0.001)	0.03*** (0.003)	−0.01 (0.18)	0.01 (0.43)	0.01 (0.20)
	Lab	0.09*** (<0.001)	0.04** (0.01)	0.06*** (0.003)	−0.001 (0.96)	0.01 (0.60)

The table shows regression coefficients and *P*-values in parentheses. For Experiment 1, we fitted a random-intercept logistic regression model for the choice of the higher-value target. Regressors included the value difference between the targets and the average values of the context items surrounding the higher-value target and lower-value target. The table shows the coefficients for the regressor representing the average context value. A positive coefficient means that the probability of choosing the higher-value target increases as the mean value of context increases. For Experiment 2, we ran a random-intercept linear regression for the WTP in the Context-BDM. Regressors were the original WTP of the target and the average of the original WTP for the context items. The table shows the coefficients for the regressor representing the average context value. A positive coefficient indicates that WTP increases as the context value increases. To quantify how the context effect changes as a function of RT, we ran the above regressions in five RT bins. RT bins were determined by quantiles (0.2, 0.4, 0.6, 0.8) of each trial type's RT. The first RT bin includes responses faster than the 0.2 quantile of RT of a trial type, whereas the fifth RT bin includes responses slower than 0.8 quantile of RT of a trial type. *P < 0.05, **P < 0.01, ***P < 0.001.

We test two hypotheses about context effects in the two experimental paradigms. The first hypothesis is that the presence of context products would produce a contrast effect analogous to the perceptual Ebbinghaus effect: the value of a target product will vary inversely with the value of the context products. We expected participants to value and choose a target product more often when surrounded by low-value products, as it would be perceived as relatively higher in value, compared to when surrounded by high-value products. We hypothesized that this relative valuation would occur during the evaluations of the target products.

The second hypothesis was that the context products would have an assimilation-like effect through the starting points. Because we allowed participants to observe all the products together for 4 seconds before revealing the target products, participants could form a prior expectation about their response. In the binary-choice experiment, we expected participants to form a prior belief that they should select the side with the better set of items (i.e. participants would shift their starting point towards the better set and be more likely, and faster, to choose the higher-value target when it is surrounded by high-value products than when it is surrounded by low-value products). If this starting-point bias extends to the WTP experiment, we would then expect context to alter the final valuation, rather than just generating a faster response. This would alter the bid in the direction of the context, so that a higher context would elicit a higher WTP for the same target item.

We find evidence for the second hypothesis, namely that our participants used the values of the sets, prior to knowing which was the target option, to determine the starting points for their choice and valuations of the target items. This resulted in participants placing a higher valuation on an item when it was surrounded by higher-valued context, even when that context was irrelevant at the time of their valuation. We do not find a conclusive effect of context on drift rates, though our best-fitting model of choice does include significant variation in the drift rate across participants. In particular, the fastest decisions and valuations were most affected by the surrounding context, while the slowest decisions and valuations were on average unbiased.

## Results

### Experiment 1—choice

**Fig. 1.**
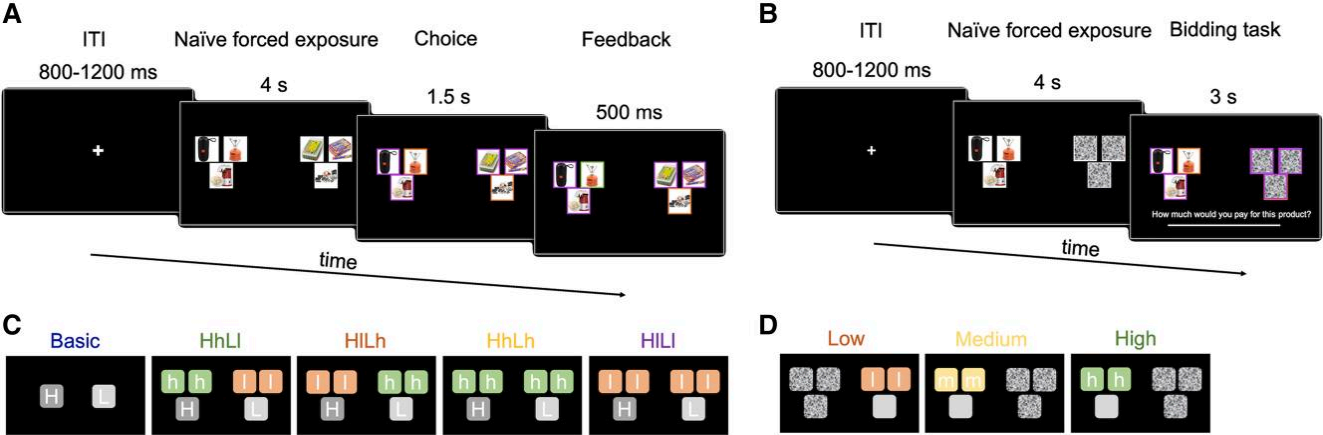
Task. Single trial timeline of (A) the binary-choice task of Experiment 1 and (B) the context-BDM task of Experiment 2. Different trial types of (C) the binary-choice task and (D) the context-BDM task. Capital letters (gray squares) represent targets. “H” and “L” represent the higher-value and the lower-value target, respectively. Lower case letters (colored squares) represent context products. The “l” (red), “m” (yellow), and “h” (green) squares represent low-, medium-, and high-context products, respectively.

All 24 participants performed two tasks. First, participants performed a standard BDM task (42) in which they reported their WTP for different consumer products. Then, they completed a binary-choice task in which they chose their preferred product. In the *Context* trials, participants had 4 seconds to inspect three products on each side of the screen. Then, one product from each side of the screen was highlighted in orange as the target item and participants had 1.5 seconds to make a choice between the two targets (Fig. 1A and C).

#### Positive effect of context on choice

**Fig. 2.**
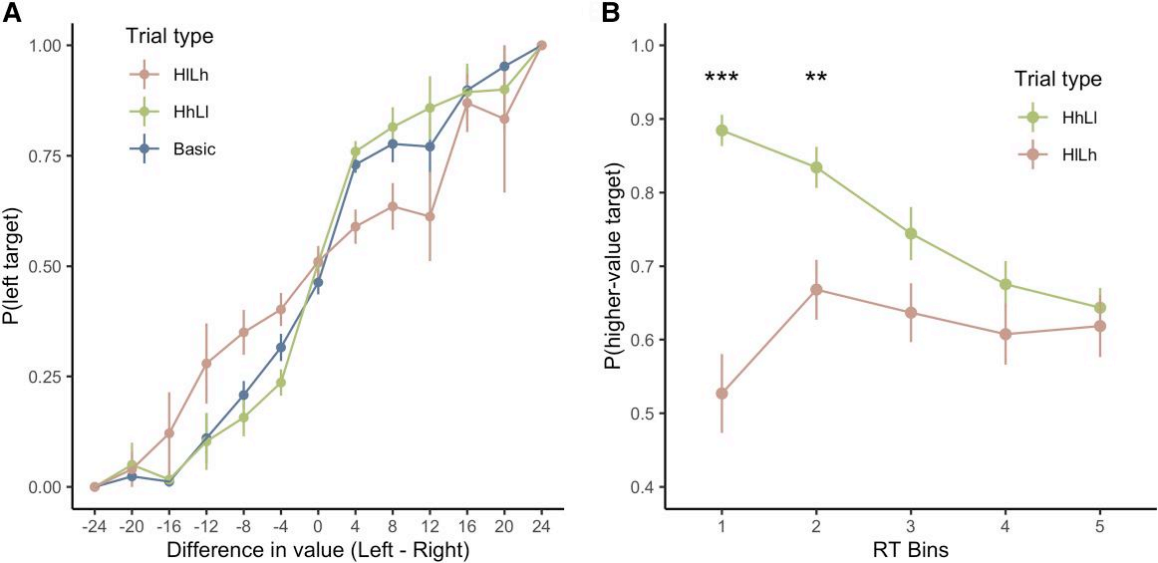
Positive effect of context on choice. (A) The probability of choosing the left target product as a function of value difference between the two target products in the Basic condition and in two main trial types (HhLl, HlLh) of the Context condition. Each dot represents the mean choice probability averaged across participants. The error bars represent standard errors across participants. (B) Probability of choosing the higher-value target as a function of RT bins. Dots represent mean choice probability averaged across participants. Error bars represent standard errors across participants. *P < 0.05, ***P < 0.001.

To examine the influence of the context products on participants’ choices, we conducted two different analyses. First, we examined how the difference in WTP between the two target products and the values of the context items affected participants’ choices relative to the *Basic* condition with no context items. We fit a random-intercept logistic regression model (Supplementary Material Appendix, Table S1; Fig. 2A). The value difference between the target products significantly affected participants’ choices (β = 0.14, *P* < 0.001). The larger the difference between the target products, the higher the choice proportion for the higher-value target. This demonstrates that participants were sensitive to the values of the target products.

Regarding the values of the context items, we also observed a significant positive effect of context. The probability of choosing the higher-value target was significantly greater when it was surrounded by high-context products (trial type HhLl: β = 0.22, *P* = 0.005; Supplementary Material Appendix, Table S1; Fig. 2A). Additionally, the probability of choosing the higher-value target was significantly lower when it was surrounded by low-context products (trial type HlLh: β = −0.54, *P* < 0.001; Supplementary Material Appendix, Table S1; Fig. 2A). Moreover, when both alternatives were surrounded by similar contexts (both sides were low-context (HlLl) or high-context (HhLh)), there was no significant effect of context on participants’ choices (Supplementary Material Appendix, Table S1).

Second, to directly assess the effect of context value, we replaced the categorical trial type variable with two parametric variables: the average values of the context surrounding the higher-value target, and lower-value target, respectively. Again, we fit a random-intercept logistic regression model to the choice of the higher-value target. Consistent with our first analysis, the probability of choosing the higher-value target increased as the mean value of context products surrounding the higher-value target increased (context mean of the higher-value target: β = 0.02, *P* < 0.001; Supplementary Material Appendix, Table S2). Additionally, the probability of choosing the higher-value target decreased as the context value mean of the lower-valued target increased (context mean of the lower-value target: β = −0.02, *P* < 0.001; Supplementary Material Appendix, Table S2). This again demonstrates what looks like a significant assimilation effect.

#### The effect of context on RT

Previous studies have shown a significant positive correlation where participants tend to choose faster when the value difference between the options is larger (15, 43, 44). We therefore examined whether there was a difference in RT between the experiment's conditions. We measure RT from the end of the naïve forced-exposure stage (when the target was highlighted) until the button-press. We expected RT in the *Basic* condition to be lower than in the *Context* condition since it would be easier to choose between two targets when they are displayed without any context products. Additionally, we hypothesized that the negative correlation between value difference and RT would be stronger and weaker when the higher-value target was surrounded by high-value context (HhLh) and low-value context (HlLh), respectively (which corresponds to the assimilation or starting-point bias hypotheses).

We examined the influence of the context products on RT with a random-intercept linear regression model on the difference in value between the two target products, the context trial type, and their interactions (Supplementary Material Appendix, Table S3, Fig. S2A). We log transformed RT after excluding responses faster than 100 ms because the RT distribution was right skewed. As expected, the absolute value difference between target products significantly decreased participants’ RT (β = −0.01, *P* < 0.001; Supplementary Material Appendix, Table S3, blue line in Fig. S2A). Meanwhile, all *Context* trial types had significant positive coefficients, meaning that participants took longer time to choose in all the *Context* trial types compared to the *Basic* condition (Supplementary Material Appendix, Table S3, Fig. S2B). This suggests that participants found *Context* trials, in general, to be more difficult.

In line with our hypothesis about the effect of different contexts on RT, in the HhLl trial type there was a significantly larger decrease in RT due to target value difference (Trial type HhLl: Absolute value difference: β = −0.01, *P* = 0.02; Supplementary Material Appendix, Table S3, green line in Fig. S2A). Meanwhile, in the HlLh trial type, there was a significantly smaller decrease in RT due to target value difference (Trial type HlLh: Absolute value difference: β = 0.01, *P* < 0.001; Supplementary Material Appendix, Table S3, red line in Fig. S2A).

Additionally, none of the control trial types (HhLh and HlLl) differed from the *Basic* condition in terms of how the target absolute value difference affected RT (Supplementary Material Appendix, Table S3). This again indicates that the significant interaction effect of the HhLl and HlLh trial types compared to the *Basic* condition is not merely due to the presence of context but rather the context value difference between the two sides.

#### Choice-RT relationship

We next tested our starting-point hypothesis, namely that the starting point would be shifted toward the boundary of the side with the higher-valued set. If the only effect of context was on the starting point, the effect on choice would only appear in quick decisions (37, 40).

We tested this prediction by plotting the choice probability as a function of RT. We computed the probability of choosing the higher-value target in the main trial types (HhLl and HlLh) in five RT bins. RT bins were determined by quantiles (0.2, 0.4, 0.6, 0.8) of each trial type's RT. For example, the first bin included all decisions faster than the 0.2 quantile of RT of a trial type, the second bin contained decisions faster than the 0.4 quantile and slower than the 0.2 quantile of RT of the trial type, etc. We then compared the choice probabilities between HhLl and HlLh with paired t-tests in each RT bin.

The probabilities of choosing the higher-value targets only differed in the first and second RT bins (Supplementary Material Appendix, Table S4; Fig. 2B). For quick decisions, participants chose the higher-value target more when it was surrounded by high-value products than when it was surrounded by low-value products. However, the choice probabilities were not different when decisions were made more slowly. These results are indicative of a starting-point effect.

#### DDM results

Among the six models considered, the best-fitting DDM included context effects on both starting point and drift rate (BPIC = 5925.23; see Supplementary Material Appendix, Table S5 for BPIC of all six models). The largest improvement in fit was due to context effects on starting point (ΔBPIC = −171.56; Supplementary Material Appendix, Table S6), though the improvement due to context effects on drift was credible (ΔBPIC = −76.07; Supplementary Material Appendix, Table S6).

**Fig. 3.**
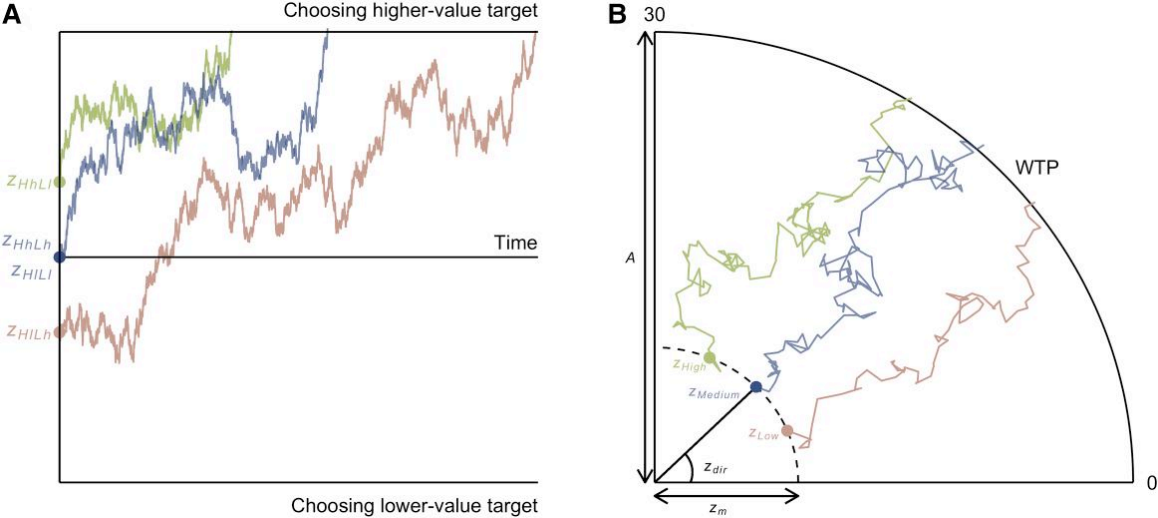
Illustration of context effect on starting point in the DDM (A) and WTP accumulation model (B). Each trajectory represents a hypothetical evidence accumulation process in a single trial. Colored dots represent the starting point of each trial type. (A) The x-axis represents the time, and the y-axis represents the evidence state. Our modeling results showed that the starting point was shifted toward the side with the higher overall value of the set of three items. This result means that the starting point in the HhLl trial type moved toward the boundary of choosing the higher-value target. (B) Coordinate in a two-dimensional space represents the evidence state. The starting point (colored dots along the dashed line) is set between the origin and boundary (the edge of a quarter circle). The magnitude $z_{m}$ and the direction $z_{dir}$ jointly determine the starting point. We assumed that the direction of the starting point would depend on the average value of the three products. Thus, $z_{dir}$ was largest in the High-context condition and smallest in Low-context condition.

The highest density interval (HDI) of the group level $z_{1}$ posterior distribution was strictly positive, i.e. the probability of $z_{1}$ above zero was 1 (Supplementary Material Appendix, Table S7). Ten out of 24 participants had a strictly positive HDI for $z_{1}$ and the mean probability of $z_{1}$ above zero was 0.76 (Supplementary Material Appendix, Table S8). A positive $z_{1}$ indicates that the starting point was shifted towards the side with the higher overall value of the set of three items (Fig. 3A). This result indicates that participants were very likely expecting to choose target products surrounded by higher context products.

The value of the target products was the main determinant of drift rate. The HDI of the group level $v_{1}$ posterior distribution (the effect of the target value difference) was strictly positive, i.e. the probability of $v_{1}$ above zero was 1 (Supplementary Material Appendix, Table S7). Twenty out of 24 participants had a strictly positive HDI and the mean probability of $v_{1}$ above zero was 0.94 (Supplementary Material Appendix, Table S8).

Context had a more variable effect on drift rate. The HDI of the group level $v_{2}$ posterior distribution (the effect of the overall value of the context products across all trial types) was not strictly positive or negative (Supplementary Material Appendix, Table S7). The probability of $v_{2}$ above zero was 0.85.

However, the direction of influence was not consistent across participants. The mean probability of $v_{2}$ above zero across participants was 0.57 (Supplementary Material Appendix, Table S8). These results suggest context had a differential effect on evidence accumulation process for each participant, although the overall effect of context on drift rate was positive.

### Experiment 2—WTP

As in Experiment 1, participants first reported their WTP for various consumer products. Next, they completed a second WTP task where they first had 4 seconds to inspect three products on the screen before the target was highlighted in orange (Fig. 1B). They were given 3 seconds to place a bid for the target item. The other context items had either *Low*, *Medium*, or *High* values (Fig. 1D). There were two samples for this experiment, one in the lab (N = 28) and one online (N = 101).

#### Positive effect of context on valuation

To examine the influence of context on WTP, we conducted two different analyses. First, we compared the bids offered in the *High* and *Low* trial types to the bids offered in the *Medium* trial type with a random-intercept linear regression model (Supplementary Material Appendix, Table S9, Fig. S4). We used the *Medium* trial type as our baseline for comparison and not the Original-BDM, since the Original-BDM and the Context-BDM differed in the amount of time given to participants to evaluate the target (Original-BDM: up to 7 seconds, Context-BDM: up to 3 seconds (in the first 4 seconds, participants did not know which product would be the target)).

Like Experiment 1, we observed a positive effect of the context products in both the *Low* and *High* trial types in the two samples. In both the *online-valuation* and the *lab-valuation* samples, bids significantly decreased when the target was surrounded by low-context products (*Online-valuation*: β = −0.30, *P* = 0.002; *Lab-valuation*: β = −0.48, *P* = 0.01), and significantly increased when the target was surrounded by high-context products (*Online-valuation*: β = 0.20, *P* = 0.04; *Lab-valuation*: β = 0.53, *P* = 0.006; Supplementary Material Appendix, Table S9, Fig. S4). Thus, compared to the medium-context products, the presence of high-context products increased the value of target products, while the presence of low-context products decreased the value of target products. These results are consistent with an assimilation effect.

**Fig. 4.**
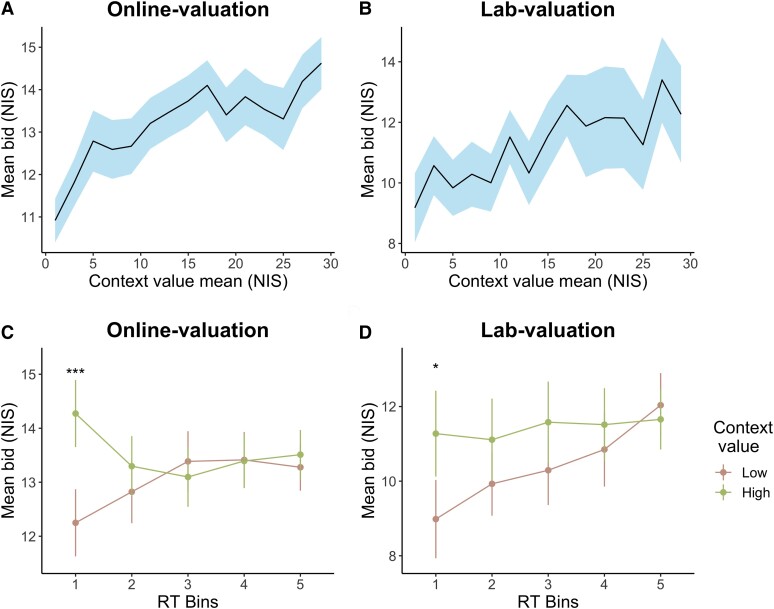
Positive context effect on participants’ valuations. (A, B) There was a significant positive relationship between the average value of context products and participants’ bids in the Context-BDM. The higher the average value of the context products was, the higher the participants’ bid was. This was evident in both *Online-valuation* and *Lab-valuation* samples. The black solid line represents the mean bids averaged across participants. The shaded area represents standard errors across participants. (C-D) Plot of mean bid within each RT quintile in the *Online*-valuation and the *Lab*-valuation samples. Each dot represents the mean bid within each RT bin. Error bars represent standard errors across participants. *P < 0.05, ***P < 0.001.

Second, to examine the effect of context value, we replaced the categorical variable of Context-BDM trial types with a parametric variable: the average of the original WTP of the context products (context value mean). Again, we fit a random-intercept linear regression model to the bids of the target products in the Context-BDM, with the original WTP for the target and context value mean as regressors. Confirming the previous result, we found that the average value of the context products had a significant positive effect on the bids in both samples (*Online-valuation*: β = 0.02, *P* < 0.001; *Lab-valuation*: β = 0.04, *P* < 0.001; Supplementary Material Appendix, Table S10, Fig. 4A-B). The higher the average value of the context products, the larger the increase in the bids for the target product in the Context-BDM, controlling for the original WTP for the target. Specifically, for each 1 NIS increase in the average value of the context products, there was a 2-cent increase in the valuation of the target product.

#### Context effects on valuation RT

As with the choice experiment, we examined whether RT was influenced by the different context trial types (*Low*, *Medium*, *High*). RT was measured from the time that the targets were highlighted until participants entered their bids. In line with the choice experiment, we hypothesized that participants would use the average value of the three products to set their starting point for the coming bid. The WTP accumulation model predicts faster decisions when the target value is closer to the average of the three products. Because the shortest route from the origin to the edge of a circle is a straight line, the fastest way to decide is to continue along the angle generated by the starting point.

To test this hypothesis, we fit a random-intercept linear regression model to the RT (in seconds) using the absolute difference between the original WTP of the target and the mean WTP of the context products as the predictor (termed “context-target absolute difference”). We did not transform RT to a log scale because the WTP–RT distributions were not skewed. In line with our hypothesis, we observed that the context-target absolute difference had a significant positive effect on RT in the online sample (*Online-valuation:* β = 0.002, *P* = 0.005; Supplementary Material Appendix, Table S11, Fig. S5A), though we did not find the effect in the lab sample (*Lab-valuation:* β = 0.001, *P* = 0.24; Supplementary Material Appendix, Table S11, Fig. S5B). This result indicates that when the target was closer in value to the context products (and hence to the set average), it was easier for participants to complete their bids.

#### WTP–RT relationship

We further tested the starting-point hypothesis by examining the relationship between WTP and RT. The previous regression result suggests that participants set expectations for the coming bid based on the context. If this was the case, bids would be shifted toward the set average value when participants did not take enough time to evaluate the target value. Specifically, bids would be higher when the target was surrounded by high-value products, and vice versa. The shift in bids would be pronounced when participants bid quickly.

We computed the mean bid of the two main trial types (*Low*, *High*) in five RT bins. As in Experiment 1, we divided the RT distribution into quintiles and within each one compared the mean bids for the two trial types (*Low*, *High*) with paired t-tests.

The mean bids for the two main trial types were different only in the first RT bin (Supplementary Material Appendix, Table S12, Fig. 4C and D). Only when participants bid quickly did they bid more for the target product when it was surrounded by high-value products than low-value products. This result also supports the starting-point hypothesis.

#### WTP accumulation model results

We simulated a WTP accumulation model (45, 46) to understand how context influences valuation. The WTP accumulation model is a DDM-like model for responses on a continuous scale. The key difference between the two models is that the WTP accumulation model assumes the evidence accumulation process occurs in a two-dimensional space (Fig. 3B). As with the DDM, the WTP accumulation model has a starting point and drift rate which determine the initial evidence state and the evidence accumulation process.

We simulated six variants of the WTP accumulation model and examined which of the six models best mimicked the behavioral data. We examined the influence of context on the bids, RT, and the relationship between the bids and the RT. First, we tested which of the models shows the observed context effect on the bids. We ran a random-intercept linear regression model on the bids. The original WTP of the target product and the mean WTP of the context products were predictors.

Among the six models, three models showed positive context effects (Supplementary Material Appendix, Table S13). The mean value of the context products had a positive effect on the bids in the *starting point* model (β = 0.02, *P* < 0.001) and the *positive drift* model (β = 0.06, *P* < 0.001). Models with *starting point* bias in addition *to positive drift* bias also showed a positive context effect (β = 0.10, *P* < 0.001). Contrary to the observed data, the *negative drift* models showed negative context effects (*negative drift*: β = −0.08, *P* < 0.001; *starting point* and *negative drift*: β = −0.03, *P* < 0.001).

Then, we examined the influence of context on RT. We ran a random-intercept linear regression model on RT. The predictor was the absolute difference between the original WTP of the target product and the mean WTP of the context products. None of the six models showed positive context effects on RT (Supplementary Material Appendix, Table S14).

**Fig. 5.**
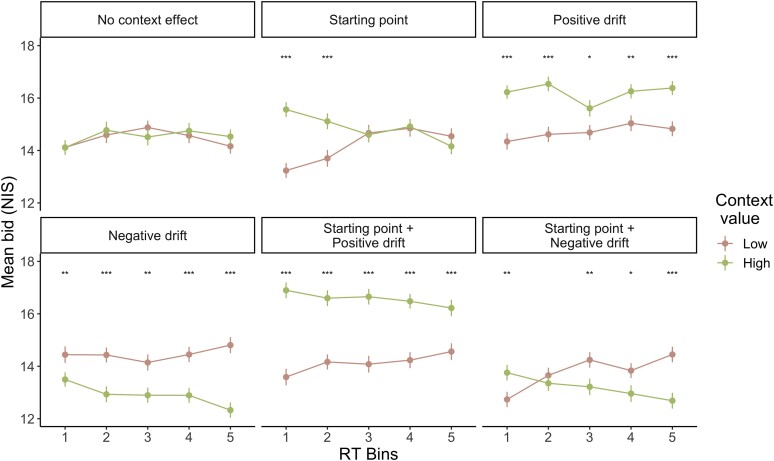
WTP accumulation model results. Simulated data showing the relationship between the mean bid and RT. Each panel shows results from each model. Each dot represents the mean bid within each RT bin averaged across participants. Error bars represent standard errors across participants. *P < 0.05, **P < 0.01, ***P < 0.001.

Lastly, we examined the relationship between the bids and RT (Supplementary Material Appendix, Table S15, Fig. 5). We computed the mean bids for the two main trial types (*Low*, *High*) in the RT quintiles and compared them with paired t-tests, as we did with the behavioral data. Among the five RT bins, the first and second RT bins were considered fast responses, and the remaining RT bins were considered slow responses.

Only the *starting point* model demonstrated the observed relationship between the bids and the RT. The *starting point* model showed a positive context effect on the bids only in fast responses. The model did not show a difference in bids for slow responses.

On the other hand, context affected the bids in fast and slow responses in the *drift bias* models. When context had a positive effect, the bids in the *High* condition were higher than in the *Low* condition in all five RT bins. The opposite was the case when the context had a negative effect.

Models with the starting-point and drift effects showed the combination of the individual effects. With starting points and a positive drift effect, context had a positive effect on the bids both in fast and slow responses. The difference between the *High* and *Low* bids was larger for fast responses than for slow responses. However, the model with starting points and a negative drift effect showed both positive and negative context effects. We observed positive context effects in the first RT bin due to the starting point and negative context effects in slow responses due to the negative drift effect.

Given the simulation results, the starting point is the most likely explanation for the positive context effect in our study. Only the *starting point* model displays the positive context effect that diminishes over time.

#### Relationship between context effect and mean RT

If indeed the context effects are primarily due to starting-point biases, we should find that time-pressure accentuates those effects (47). While we do not implement a time-pressure manipulation, we can exploit natural variation in participants’ response caution (i.e. boundary separation) as approximated by their mean RT. Pooling the data across both experiments and normalizing the regression-based context effects and RT within each experiment, we indeed found a significant negative relationship between mean RT and the size of the context effects across participants (normalized mean RT: β = −0.25, *P* = 0.002; Fig. S7; Supplementary Material Appendix for details).

## Discussion

In the current study, we aimed to better understand the effect of spatial context on WTP and choice through the distortion of prior expectations, relative evaluations, or both. In both of our experiments, we observed a consistent assimilation-like effect where contextual items had an attractive effect on the target items such that higher-valued contexts made the target items more valuable and more likely to be chosen. However, this effect was only observed for the fastest decisions; it was not significant for slower decisions. This pattern, backed up by our computational modeling, indicates that in this setting, context affects behavior largely through prior expectations rather than relative evaluations. Unavailable context affects people's prior expectations and thus informs their choices and valuations but has small and/or inconsistent effects on how people perceive the targets during choice. Hence, we were able to show that the effect of context on choice and WTP depends on whether the context products can be chosen.

Our study had several special features that allowed us to study the mechanisms of context effects. First, our context items were “phantom decoys”—they were not available to be chosen (48–50). Phantom decoys were crucial because they allowed the context products to have values higher than the target products. In typical paradigms where the context products can be chosen, the decoys must be lower in value than the target products otherwise they are likely to be chosen and thus lose the status of context. Our design allowed us to use any combination of decoys, without the risk of them being chosen.

Second, all three items in each set were available for inspection for several seconds before the targets were revealed. This is an important feature for a phantom decoy design since it encourages participants to attend to all the products. Had the targets been revealed from the start, our participants might have simply focused on those products and ignored the others. An additional benefit of this design is that it allowed us to determine whether context effects are due to distorted expectations or evaluations, by exaggerating their temporal dissociation. With several seconds to form expectations about the value of each set, participants could form clear expectations before the targets were identified.

Third, and perhaps most importantly, we conducted both binary-choice and WTP experiments. By doing so, we were able to establish that context effects are not unique to choice, and that prior expectations (i.e. starting points) are not simply response biases (also see (35)). Rather, we demonstrated that subjects are willing to bid more for the same item when it has previously been associated with a high context, even when that context is no longer available. Starting points are often thought to be due to motor preparation favoring one action over another (40). However, in our valuation study, participants had to move a sliding bar to settle on their desired WTP. We still observed that higher context items led participants to bid more for faster responses, while for slower responses the role of context diminished. This is a telltale sign of prior expectations influencing behavior.

Our results also emphasize that there are critical differences between perceptual and value-based decision processes. Our initial hypothesis was that we would observe a contrast effect analogous to the perceptual Ebbinghaus effect. In perceptual choice, even if the context items are irrelevant to the task (e.g. Ebbinghaus illusion), there is a robust contrast effect. However, we did not observe a contrast effect in our value-based choice task. This might indicate that in order for the context items to be considered and have any influence over the decision, they must be available to choose (14, 51, 52). Perhaps more neural resources are required to represent the value of a stimulus compared to its perceptual properties, causing irrelevant stimuli to be more easily discarded in value-based tasks compared to perceptual tasks. This notion is in line with previous studies which posit that valuation is hard (53) and that there needs to be sufficient motivation in order for the brain to conduct a valuation process (54; but see 55, 56).

In line with past research (57–59), we observed lagged trial effects where the value of the target item in the previous trial had an assimilation effect on the current trial's target value. Also, the previous trial's context items had a small contrast effect on the current trial's target value. These results are detailed in the Supplementary Material. A potential explanation for the positive effect of the previous trial is a bias in the starting point (57). The accumulator associated with the response from the previous trial might gain an advantage in the starting point in the next trial, not having fully reset to baseline.

There are many settings in life where people are exposed to information before being confronted with a choice, and thus have an opportunity to use initial valuations to inform their decisions. Before evaluating a house, one might form an expectation based on the neighborhood. Before evaluating a television, one might form an expectation based on the other TVs available at the retailer. Before evaluating an entrée, one might form an expectation based on the other items on the menu. There are also settings in which some items become unavailable at the time of choice, such as when a retailer sells out of a high-value product. If evaluations were perfect, we would discard this contextual information and only consider the target/available items. But evaluations are not perfect, they are noisy and time consuming, and so contextual information can be useful. Like heuristics, using contextual information is generally adaptive, but in artificial settings like ours, its weaknesses can be exposed. When the context is substantially different from the target, relying too heavily on the context can lead to large mistakes, especially under time pressure (47). For example, our results would suggest that the presentation of high-value, sold-out items would increase the WTP for the remaining items.

While our conclusion on the presence of a starting-point bias is clear, we are less conclusive on the form or presence of relative evaluation in this setting. In the choice experiment, our computational modeling does suggest a distribution of relative evaluation (drift) effects across the participant sample and rejects a null effect in the population. In our main trial types, the posterior density across the sample suggests the effect of context is likely positive (i.e. an assimilation effect). However, the variation across participants is also large. In the WTP experiment, a collapsing pattern of bids across RT (i.e. no difference for longer RT) is inconsistent with an assimilation effect of context, and the lack of a reversed difference at the longest RT is inconsistent with a contrast effect of context. This suggests that these two mechanisms might be operating in parallel, with the starting point bias more consequential in magnitude.

This variation across participants and experimental paradigms is consistent with several recent studies on the influence of context on choice behavior, focusing on the effect of seemingly “irrelevant” choice alternatives. In a design where all items can be chosen, Louie et al. (13) report that the relative probability of choosing the highest-value product from the choice set is lower in the presence of higher-value alternatives compared to lower-value alternatives—consistent with a contrast effect in perception. While this effect is observed on average, there is variation across subjects (see (15) and replies by Webb et al. (16) and Gluth et al. (17)). In the domain of lotteries, Chau and colleagues use a “naïve forced-exposure” design in which all lotteries are initially presented to the participant, but one distractor lottery is declared to be “unavailable” 0.1 seconds into the trial (60, 61). Unlike our study, the context alternatives can be chosen, but are not rewarded, and are not specifically associated with a target alternative. Like us, they find an assimilation-like effect in which high-value distractors increase the probability of choosing a higher-value alternative, particularly on harder trials. However, Cao and Tsetsos (62) find several possible confounds in these studies (60, 61) and after reanalyzing the data and controlling for the confounds, report a weak contrast effect. In our design, the items were presented for much longer (4 seconds) before the target option was indicated.

Finally, previous studies on efficient coding have examined choice effects while explicitly varying the prior distribution of stimuli over many trials (5, 63), which we did not do here. Therefore, our conclusions should only be considered to apply to relatively short manipulations of prior (unavailable) context.

## Materials and methods

### Experiment 1—choice

#### Participants

A total of 28 participants (18 women, 10 men; age: range = 19–32 years, *M* = 22.18, *SD* = 2.61) participated in Experiment 1. We excluded four participants from the analysis who valued more than 25% of products under 2 NIS because we could not present them with adequate choice alternatives. The experiment was conducted in the laboratory on monitors with screen resolutions of 1,920 × 1,080 pixels. All participants received credit or a participation fee and were also paid according to their winnings in the experiment. They all provided written informed consent. The study was approved by the ethics committee at Tel Aviv University.

#### Experimental design

Both the BDM and choice tasks were preceded by a short training phase, where participants had five trials to get acquainted with the task interface. See Supplementary Material appendix for additional details.

##### BDM task

The BDM task was adapted from the standard BDM task (42). Participants received 30 NIS in the beginning of the experiment to bid for the items presented to them. In each trial, participants observed an image of a product with a short text description of the product above the image. They indicated the maximal amount they were willing to pay for the product (WTP) between 0 and 30 NIS (∼10$). They adjusted the cursor to their intended bid amount using the mouse and confirmed their bid with a single mouse click. Importantly, the initiation of the cursor in each trial was randomized across the sliding bar. If the participant did not respond within 7 seconds, we dropped this trial from the mean bid calculation. At the end of the experiment, one of the trials was chosen and a random number between 0 and 30 were chosen. If the participant bid higher for that item compared to the chosen random number, they won the item. If not, they kept the 30 NIS. The task included 60 products. Each product was presented three times in a random order. We used the average value of the three bids as the basic value of the item.

##### Binary-choice task

The choice task had two conditions (Fig. 1A). In the *Basic* condition participants saw one product on each side of the screen, while in the *Context* condition they saw three products on each side of the screen.

In both conditions, participants were instructed to observe all products on the screen for the first 4 seconds (naïve forced exposure). During this time, they were not able to make their choice. Notably, in the *Context* condition, they did not know which of the three products on each side would be available for choice. After the 4 seconds, the target product on each side of the screen was highlighted with an orange-colored bounding box. In the *Context* condition, the remaining context products were highlighted with purple-colored bounding boxes.

Participants had up to 1.5 seconds to choose their preferred target product by clicking the mouse. Thereafter, the chosen target was highlighted by a green-colored bounding box. If a participant did not make a choice in the given time, a message indicating a mistrial appeared on the screen.

The *Context* condition had four trial types. Trial types differed in the value of the products presented as context (Fig. 1C). The higher-value target and the lower-value target were surrounded by two high-context products and two low-context products, respectively (HhLl), or two low-context products and two high-context products, respectively (HlLh), or all four context products were high value (HhLh) or low value (HlLl). The last two trial types were added as controls to examine whether having different context values surrounding the targets is critical for context effects.

There were 60 trials for each main trial type (HhLl and HlLh) and 30 trials for each control trial type (HhLh and HlLl), resulting in 180 trials in the *Context* condition. There were 120 trials in the *Basic* condition. See Supplementary Material Appendix for additional details on how the target and context products were chosen.

#### Drift-diffusion model

The DDM (34) was used to account for choices and RT from the four trial types of the *Context* condition. Before the targets were revealed, it is possible that participants started to evaluate the products and developed a belief about the value of the two sides. Because this evaluation would technically occur before the beginning of the actual decision process, it would affect the starting point in the DDM.

We constructed three models for how context might influence the starting point (see Supplementary Material Appendix for additional details on hypotheses). In the baseline model, the starting point is constrained to be 0.5. In the target-only model, the value of the target products determines the starting point: $z_{i}∼z_{0\;}+z_{1}\Delta x_{target,i}$. Here $\Delta x_{target,i}$ represents the value difference between the two target products in trial $i:\Delta x_{target,i}\;=\;x_{higher-value\;target,i}-x_{lower-value\;target,i}$. *x* represents the mean bid for the product in the BDM task. In the context model, the value of all products determines the starting point: $z_{i}∼z_{0}+z_{1}\Delta x_{side,i}$. Here $\Delta x_{side,i}$ represents the difference between the summed value of the three products on each side of trial $i:\Delta x_{side,i}\;=\;\sum x_{higher-value\;target\;side,i}-\sum x_{lower-value\;target\;side,i}$.

Besides starting-point effects, nontarget products might continue to influence the evidence accumulation process after the targets are revealed. The nontarget products could either enhance (assimilation effect) or suppress (contrast effect) the evidence for their associated target products. This would be reflected in the drift rate of the DDM.

We constructed two models to test the influence of context on the evidence accumulation process. In the target-only model, only the values of the targets determine the drift rate: $v_{i}=\;v_{0}+v_{1}\Delta x_{target,i}$. In the context model, the values of all products linearly determine the drift rate: $v_{i}=v_{0}+v_{1}\Delta x_{target,i}+v_{2}\Delta x_{context,i}$. Here, $\Delta x_{context,i}$ represents the difference between the summed value of the two nontarget groups of products in the trial.

We tested these combinations of context effects on starting point and drift rate, resulting in six models (Supplementary Material Appendix, Table S5). We fitted the choice and RT data to models using HDDM (64). The performance of the models was compared using the Bayesian Predictive Information Criterion (BPIC). The model with the lowest BPIC was selected as the best model. See Supplementary Material Appendix for details on model fitting procedure and model fits.

### Experiment 2—WTP

#### Participants

One hundred and forty-four participants completed the *Online-valuation* experiment (63 women, 81 men; age: range =19–34 years, *M* = 23.35, *SD* = 2.58). Additionally, 38 participants (22 women, 16 men; age: range = 19–48 years, *M* = 25.73, *SD* = 7.14) completed the *Lab-valuation* experiment. The only difference between the two experiments was that the lab experiment was incentive compatible, while the online experiment was not (participants were given course credits for participating). We excluded 43 participants from the online experiment and 10 participants from the lab experiment (Supplementary Material Appendix).

#### Experimental design

##### Original-BDM task

The original-BDM task resembled the one in Experiment 1 except for the number of repetitions per product. Each of the 60 products was repeated twice (resulting in 120 trials), in a random order.

##### Context-BDM task

The Context-BDM task involved another BDM auction. This time the target product was surrounded by other products serving as context (Fig. 1B). In each trial, participants saw three products on one side and three images of white noise on the other side. The sides were counterbalanced across trials. This presentation was chosen to resemble the binary-choice task of Experiment 1 as much as possible.

For the first 4 seconds of a trial, participants observed the three products without knowing which one of them would be the target product (naïve forced exposure). After 4 seconds, one product was highlighted with an orange-colored bounding box, indicating the target product. The other two products were highlighted with purple-colored bounding boxes, indicating the context products. The three images of white noise were also highlighted with purple-colored bounding boxes. A sliding bar appeared below the images once the target product was revealed. Participants indicated how much they would be willing to pay for the target product between 0 and 30 NIS within 3 seconds. As in the Original-BDM task, the initial position of a cursor was randomized across the sliding bar. Participants moved the cursor to their intended bid amount and confirmed their bid with a single mouse click.

The Context-BDM task had three trial types (Fig. 1D). The target was surrounded by two high-context products (*High* context), two low-context products (*Low* context), or two medium-context products (*Medium* context). The latter served as a control for the *Low* and *High* trial types. Each of the 20 target products was presented twice in each trial type, resulting in 120 trials in total. See Supplementary Material Appendix for additional details.

#### WTP accumulation model

We simulated a WTP accumulation model (Fig. 3B) (45, 46) to understand how context influences valuation. Context could influence valuation through two mechanisms in our study. First, context could influence the starting point. Participants could begin to evaluate the three products before knowing the target product. This initial evaluation of the set could form a prior expectation for the upcoming bid on the target product. This prior expectation would be captured by the starting point.

We constructed two models examining the effect of context on the starting point. In the *baseline* model, context does not affect the starting point, which is set at the origin. In the *starting point* model, the average value of the three products determines the direction of the starting point: $z_{dir,i}∼z_{0}+z_{1}WTP_{average,i}$. The magnitude of the starting point is set to be above zero. Here $WTP_{average,i}$ is the average value of the three products in trial *i*.

Second, context could influence valuation through the drift. After the target is revealed, context products should become irrelevant. However, they could still affect the valuation of the target product. The context products could either positively (assimilation effect) or negatively (contrast effect) affect the WTP of the target product through the drift.

We constructed two models to examine the effect of context on the drift. In the *baseline* model, context does not affect the drift. Only the value of the target determines the direction of the drift: $v_{dir,i}∼v_{0}+v_{1}WTP_{target,i}$. In the *drift* model, the value of the target product and the context products determine the direction of the drift: $v_{dir,i}∼v_{0}+v_{1}WTP_{target,i}+v_{2}WTP_{context,i}$. Context could have a positive or negative effect on the drift depending on the value of $v_{2}$. We considered positive or negative effects of context on drift as two separate models. Here $WTP_{target,i}$ represents the value of the target product and $WTP_{context,i}$ represents the average value of the context products.

We considered all combinations of the above-mentioned context effects on the starting point and the drift. We simulated six variants of the WTP accumulation model to generate bids and RT data in the Context-BDM task. Then, we examined which of the six models best mimicked the bids and RT data in the Context-BDM task. See Supplementary Material Appendix for additional details of the WTP accumulation model and simulation procedure.

## Supplementary Material

pgae232_Supplementary_Data

## Data Availability

All data and code used in the analysis are available at OSF can be accessed at https://osf.io/ywa37/ (65).
