# Supplementary material for: Valuations of target items are drawn towards unavailable decoy items due to prior expectations
Source: PNAS Nexus. 2024 Jun 24;3(7):pgae232. doi: 10.1093/pnasnexus/pgae232 (PMC11214102; doi:10.1093/pnasnexus/pgae232)
Supplement: pgae232_Supplementary_Data [file pgae232_supplementary_data.docx]

**
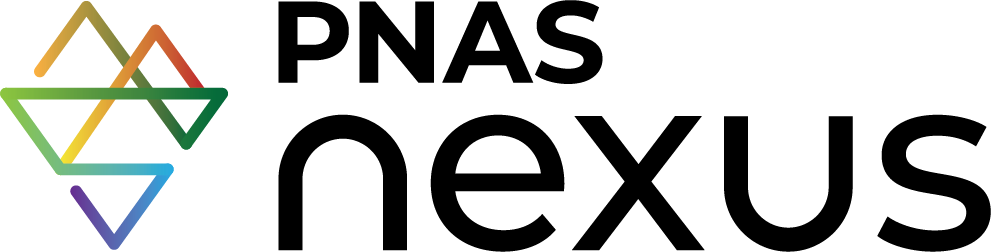
**

**Supplementary Information for**

Valuations of target items are drawn towards unavailable decoy items due to prior expectations.

Liz Izakson, Minhee Yoo, Adam Hakim, Ian Krajbich, Ryan Webb, Dino J Levy

Dino J Levy

Email: dinolevy@tauex.tau.ac.il

**This PDF file includes:**

Supplementary text

Figures S1 to S7

Tables S1 to S19

SI References

**Supplementary Text**

**Experiment 1 – Choice**

**Materials and Methods**

**Experimental Design**

***Stimuli*.** There were 75 products in the experiment. Fifteen of them appeared only on the training trials, while the remaining 60 were selected for the experiment trials, both for the BDM and the binary-choice tasks. The products were from 5 categories: electrical appliances, office utensils, food products, kitchen tools, and traveling equipment. There were 12 products in each category. To encourage participants to bid on the products according to their personal preferences, all products’ real-world prices were between 50 to 100 NIS (*M* = 69.93, *SD* = 16.55), which are well above the maximal bid price. We cropped the images of the products directly from the websites from which they were sold, added white background to achieve similar proportions between the product and its background, and then rescaled the images to the same exact size. Finally, we adjusted the images to equalize their brightness and contrast across all products to balance all possible low-level features of the product images.

***Target and context products in the binary-choice task.*** Based on the BDM task, we ranked the 60 products from the least to most valued, for each participant separately. We ranked the products based on their mean bids, averaged across the three repetitions of each product. We used the 10 lowest-ranked products and the 10 highest-ranked products as the “low-context” and the “high-context” products, respectively, in the following binary-choice task. In each trial that included context products (*Context* condition), the context products were randomly chosen from the “low-context” or “high-context” products. We used only the remaining 40 “middle value” products as target products available for choosing.

We chose the target products based on a predetermined distribution of ranks (SI Appendix, Fig. S1). In the Basic condition, the number of appearances of a target product was based on the frequency of appearance predetermined for its rank. For example, the product with rank 20 was shown 10 times in Basic condition, each time with a different product.

In the Context condition, the distribution was constructed such that all target ranks, from 10 to 50, would appear in the experiment in approximately the same frequency, while slightly emphasizing mid-range products (20-40), where decisions were likely harder and possibly more prone to context effects (1). Additionally, we predetermined the difference between the ranks of the two target products, such that differences appeared approximately in an equal amount for the entire range of ranks. For example, a difference of 10 ranks would occur in a trial for target products that are ranked 10 and 20, but also for target products that are ranked 40 and 50, with similar frequency. The average of rank differences between each two target products across all trials was 11.2, with a standard deviation of 6.9, and some positive skewness (1.06) towards choosing lower differences more often than larger differences.

***Rewards.*** After the completion of both tasks (BDM and binary-choice), one trial of the total 480 trials was chosen at random to be implemented, making the experiment incentive compatible. If the chosen trial was from the BDM task, the computer randomly generated a price for the product of the chosen trial, between 0 to 30 NIS. If the amount the participant offered for the product in that trial was larger than the random price, the participant received the product at the random price and kept the remaining money from their endowment. If the random price was larger than the amount the participant offered, then the participant did not win the product and kept the endowment in its entirety (30 NIS). If the randomly chosen trial was from the binary-choice task, the participant won the chosen product in that trial and kept the total endowment (30 NIS).

***Attention* *trials*.** We added *attention* trials in various places during the binary-choice task to make sure participants paid attention to all the products that were presented on the screen – both context and target. We added the *attention* trials based on the design used in a previous study (2). Thirteen of the 24 participants (after excluding 4 participants) had 39 additional *attention* trials embedded throughout the binary-choice task. Twelve *attention* trials were shown in the *Basic* condition, and 27 trials were shown in the *Context* condition. When an *attention* trial followed a choice trial, a single product appeared on the screen and the participant had to respond whether this product appeared on the right or left side of the screen in the previous choice trial, by clicking on the corresponding button on the mouse. For every correct answer, the participant earned an additional 0.5 NIS.

**DDM.**

The DDM (3) was used to account for choices and RT from the four trial types of the *Context* condition of the binary-choice task. The model assumes that the process of choosing between two options is based on accumulating evidence for one option relative to the other option. A decision is made once the accumulated evidence reaches one of the two decision boundaries. Choices and RT are determined by where the evidence starts to accumulate (starting point $z$) and how fast the evidence accumulates (drift rate $v$).

We considered three hypotheses about how context might influence the starting point. First, it is possible that no evaluation occurs before the targets are identified. In this case, the starting point would be halfway between the two choice boundaries. Second, it is possible that evaluation occurs before the targets are identified but that this occurs for each product separately. In this case, it is possible that only the evidence for the target products would be retained and this would determine the starting point. In this case, the starting point would be closer to the boundary for the higher-value target. Third, it is possible that a combined evaluation occurs for all products in a set before the targets are revealed and this combined evaluation affects the starting point. In this case, the starting point would be closer to the boundary for the higher value three-item set. This third hypothesis predicts a difference in the starting point between HhLl and HlLh trial types.

Models were fit to the choice and RT data using HDDM, a Python package for fitting the DDM with a hierarchical Bayesian estimation approach (4). Three chains were used in each model. Each chain had 6000 samples after discarding 4000 samples as burn-in. $\hat{R}$ values of all parameters for all models were smaller than 1.1, indicating successful convergence between chains.

The performance of the models was compared using the Bayesian Predictive Information Criterion (BPIC). The model with the lowest BPIC was selected as the best model (SI Appendix, Table S5). To check how well the best-fitting model accounts for choices and RT, we simulated it with 500 samples from the posterior distributions of its parameter values. This simulated data was compared with the observed data. See Fig. S3 for model fits.

**Results**

**Asymmetry in the effect of low- vs. high- value contexts.** We examined whether there was an asymmetry in the size of the effect between the low and high contexts. We compared the HhLl (β = 0.22) and HlLh (β = -0.54) coefficients (in absolute value) and found an asymmetry in the effect size of low- and high- contexts. Low-context produced a significantly larger effect on participants’ choices in comparison with high-context (difference in absolute value = 0.32, $\chi^{2}$ = 6.80, p = .009).

**Experiment 2 – WTP**

**Materials and Methods**

**Participant exclusion criteria.** To ensure that the online participants were engaged in the task, we defined several exclusion criteria prior to analyzing the data: more than 25% of products valued under 2 NIS, less than 0.75 correlation between the original-BDM task, mean RT less than 1500 ms in the original-BDM task, and more than 20% of missed trials in the context-BDM task. We chose these exclusion criteria after examining the distribution of each of the corresponding parameters; these criteria separated the outliers well from the rest of the distribution (above 3 standard deviations difference from the mean of the distribution). These criteria excluded 43 participants from the online experiment and 10 participants from the lab experiment. After defining these criteria, we also verified that all participants in Experiment 1 satisfied them.

**Experimental Design**

***Stimuli.*** For each participant, products were ranked according to the average WTP values (across the two repetitions) from the original-BDM task. The 10 lowest-ranked products were used as the “low-context”, and the 10 highest-ranked products as the “high-context”. These 20 products were not presented as target products for evaluation. The next 5 highest (ranks 46-50) and 5 lowest (ranks 11-15) ranked products were discarded from the experiment to make target products more distinct from the context products, leaving 30 medium-ranked products (ranks 16-45). Out of these 30, we randomly chose 10 products which served as “medium-context” products. These products were also excluded from appearing as targets. Only the remaining 20 products were used as target products and were presented during the Context-BDM task. In each trial of the Context-BDM task, the context products were chosen at random out of these three pools of context products: “low-context”, “medium-context”, and “high-context”.

***Catch trials*.** We added 20 *catch* trials in the Context-BDM task. The target product was shown as a context product (via a purple bounding-box), and a random context product was presented as the target product (via an orange bounding-box). In a *catch* trial the target product was either a low- or a high-context product, and the surrounding products (context) included a medium-ranked product that was a target product in all trial types. The purpose of these *catch* trials was to obscure the design of the experiment and prevent participants from memorizing which products were most often context and which were target.

**WTP accumulation model**. We simulated a WTP accumulation model (5, 6) to understand how context influences valuation. The WTP accumulation model is a DDM-like model for responses on a continuous scale (Fig. 3B). The model assumes that a decision maker collects noisy samples of evidence over time. The decision maker responds once enough evidence supporting a bid is collected.

We constructed the model in the first quadrant of a two-dimensional space. A two-dimensional vector $s(t) = [$ $s_{x}, s_{y}]$represents the evidence state. The angle of $s(t)$ represents the estimate of WTP at time *t*. The minimum and maximum WTP correspond to the minimum and maximum angles of the first quadrant, i.e., 0 and $\pi/2$. Any WTP can be transformed into an angle by $(WTP-{WTP}_{min})/({WTP}_{max}-{WTP}_{min})\cdot(\pi/2)$ which in our case simplifies to $(WTP$ $/$ $30)\cdot(\pi/2)$. The evidence state $s\left( t \right)$ starts at the origin $(s(0) = [0,0])$ and evolves until it reaches a predetermined distance from the origin. One can visualize this process as diffusion from the center to the edge of a quarter circle. The radius of the circle is roughly equivalent to the boundary separation in the DDM. Thus, the closer $s(t)$ is to the edge of the circle, the closer the decision maker is to making their decision.

The drift $v$ and the diffusion noise $\sigma$ determine the diffusion process. The drift is a two-dimensional vector $v = [$ $v_{x,}v_{y}]$. The drift can be expressed as an angle $v_{dir} = arctan(v_{y}/v_{x})$ and a magnitude $v_{m} =\sqrt{{v_{x}}^{2}+ {v_{y}}^{2}}$. The angle determines the average direction of the diffusion process and reflects the WTP favored by the evaluation of the item. The magnitude determines how quickly evidence is accumulated and reflects the quality of the information from the item. Diffusion noise is also a two-dimensional vector $\sigma$ $= [$ $\sigma_{x},\sigma_{y}]$.

The diffusion process was approximated with the Euler method (7) with a time step $\Delta$ $t$ of 10 ms. The evidence state is updated at each time step as follows: $s(t+$ $\Delta$ $t) = s(t) + v$ $\Delta$ $t +$ $\sigma$ $\sqrt{\Delta t}$. We fixed the standard deviation of the diffusion process to 1 for both $\sigma_{x}$and $\sigma_{y}$. Also, we assumed that the diffusion noise is independent in *x* and *y*. We used reflecting bounds to constrain the diffusion process within the first quadrant (i.e., between 0 and $\pi/2$).

A response is made when $s(t)$reaches a radius of $A$ from the origin (not necessarily the starting point). The point where $s(t)$reaches a radius of *A* determines the stated WTP as follows: $(arctan($ $s_{y}/s_{x})/(\pi/2))\cdot({WTP}_{max}-{WTP}_{min})$ which in our case simplifies to:

$(arctan($ $s_{y}/s_{x})/(\pi/2))\cdot30.$ The number of time steps to reach *A* ($n_{\Delta t}$) plus the non-decision time $ndt$ determines the RT: $n_{\Delta t}\cdot\Delta t+ndt$. Non-decision time captures non-decision-related processes such as stimulus encoding and motor preparation time.

The diffusion process need not start at the origin. Like with the starting point in the DDM, prior information may allow the decision maker to start the valuation process closer to some WTP than others. In our experiment, participants could begin to evaluate the products during the naïve forced exposure epoch, before knowing which product would later become the target. This initial evaluation of the set could form the basis for a shift in the starting point: $s(0) = z = [$ $z_{x}, z_{y}]$. As with the drift rate, the starting point can be expressed as an angle $z_{dir}$ and a magnitude $z_{m}$. The angle reflects the favored WTP just as the target is revealed and the magnitude reflects how much this initial evaluation will affect the final WTP. As the magnitude increases, the initial evidence state $s(0)$ starts closer to the choice boundary *A*.

***Simulations*.** We considered combinations of context effects on the starting point and the drift by simulating six datasets of bids and RT in the Context-BDM task. To simulate models for a participant, we used empirical value distributions of target and context products for that participant. We used the average WTP (across the two repetitions) from the original-BDM task as the values of the target and context products. We sampled one set of parameter values for the full model and removed some parameters in the constrained models. The full model was the model assuming context effect on both the starting point and the drift (either positive or negative). We sampled parameter values for a participant from the following distributions (see Fig. S6 for the distribution of parameters for simulations):

$A \sim Gamma(10, 0.2)$

$ndt \sim Gamma(1.5, 0.1)$

$z_{0}\sim Normal(0, 0.2)$

$z_{1}\sim Normal(1, 0.2)$

$z_{m} \sim Beta(4,20)$

$v_{0} \sim Normal(0, 0.2)$

$v_{1}\sim Normal(1, 0.2)$

$v_{2}\sim Normal(0.2, 0.1) for a positive context effect$

$v_{2} \sim Normal(-0.2, 0.1) for a negative context effect$

$v_{m}=1$

**Results**

**Lagged trial effects on the WTP in the Context BDM.** We looked into possible lagged effects of earlier trials’ target values and context values on the current trial’s valuation. We used a forward model selection approach in which we progressively added lagged effects to test whether those additions would improve model fit. We found that adding the previous trial’s (t-1) values improved model fit but going back further into the past did not. The previous trial’s value had an assimilation effect while the previous trial’s context had a slight contrast effect.

We analyzed the WTP data using linear regression models with the target’s WTP in the context BDM as the dependent variable and random intercepts at the subject level. In the baseline model, we included the target’s original WTP and the average context items’ WTP. Next, we progressively added information from the previous trial (t-1), the trial before that t-2, t-3, and t-4. Each time, we added the average WTP for that trial’s context items and the WTP for that trial’s target item. We used two sets of models, one using the target item’s original WTP (Table S16) and one using the target item’s WTP in the Context BDM (i.e., the actual response in the previous trial) (Table S17).

We compared these models using likelihood ratio tests and the resulting chi-squared statistics, testing whether adding an additional lagged trial improved model fit. Adding trial t-1 improved model fit (Model with the original WTP: *Online*: $\chi^{2}$(3) = 56.30, p < .001; *Lab*: $\chi^{2}$(3) = 23.47, p < .001, Table S16; Model with the actual response in the Context BDM: *Online*: $\chi^{2}$(3) = 71.91, p < .001; *Lab*: $\chi^{2}$(3) = 28.58, p < .001, Table S17). However, adding additional lagged trials did not further improve model fit (Model with the original WTP: all p > 0.08, Table S16; Model with the actual response in the Context BDM: all p > .09, Table S17).

To examine the effects of the lagged variables, we examined the regression coefficients. The target WTP in the current trial was higher when the target item in the previous trial had a higher original WTP (*Online*: β = 0.03, p = <.001; *Lab*: β = 0.01, p = .34; Table S18) or participants placed a higher bid in the previous trial (*Online*: β = 0.03, p < .001; *Lab*: β = 0.03, p = . 01; Table S19). The target WTP in the current trial was also higher when the context items in the previous trial had lower WTP (*Online*: β = -0.01, p = .03; *Lab*: β = -0.02, p = .02; Table S18; *Online*: β = -0.01, p = .02; *Lab*: β = -0.02, p = . 007; Table S19).

**Relationship between context effect and mean response time (RT).** In the DDM, time-pressure leads to reduced boundary separation, which accentuates the effects of starting-point bias (8). We did not have a time-pressure manipulation in our study. However, we can exploit the fact that participants do vary in their response caution (i.e., boundary separation). While this isn’t quite the same as an exogenous time-pressure manipulation, it is a well-established individual-difference measure. Thus, we examined whether participants with shorter average RT, across all their decisions, tend to show stronger context effects. Indeed, we find a significant negative correlation between mean RT and size of the context effects.

We measured context effects using regression coefficients. In Experiment 1, we ran a logistic regression on choice for each participant. Regressors were the target value difference, the average values of the context surrounding the higher-value target and the lower-value target (the same regression as in Table S2, SI Appendix). The context effect was defined as the difference in the two context coefficients. In Experiment 2, we ran a linear regression on bids for the target in the Context BDM for each participant. Regressors were the original WTP for the target and the mean value of the context items (the same regression as in Table S10, SI Appendix). Here the context effect was defined as the coefficient on the context values.

We pooled the data from Experiment 1 and Experiment 2 after normalizing the context effects and RT within each experiment. Then, we ran a linear regression of the normalized context effect on the normalized mean RT. The regression showed that the context effect was stronger in participants with shorter RT (normalized mean RT: β = -0.25, p = .002; Fig. S7). Note that the result remains significant after excluding one outlier subject (marked with a green triangle) with a strong positive context effect (normalized mean RT: β = -0.11, p = .01).


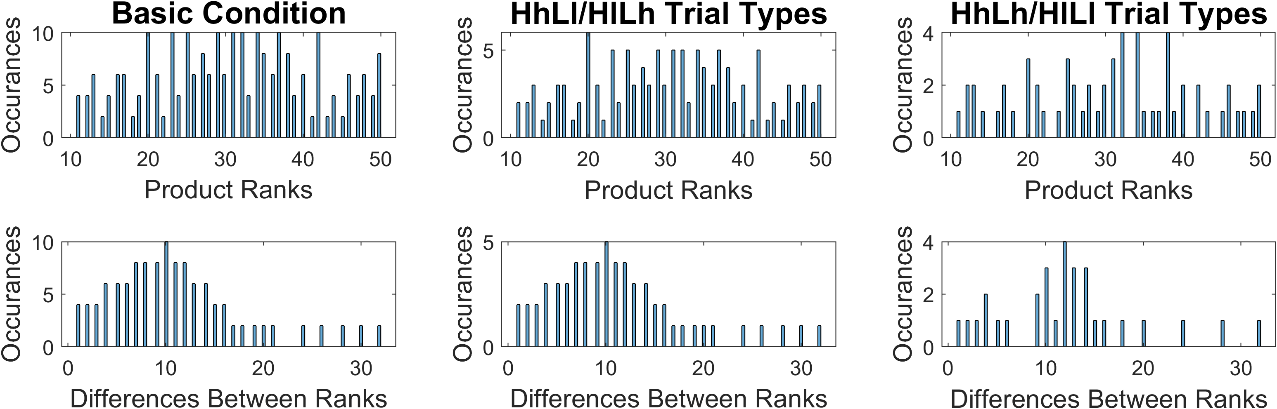


**Fig. S1.** Distribution of ranks and differences between ranks for each condition and trial type. The top row describes the number of times each product rank appeared as a target product in the *Basic* condition (left), in the HhLl and HlLh trial types (middle), and in the HhLh and HlLl trial types (right). The ranks were based on the average WTP values for each participant across all their bids. The bottom row shows the number of times each difference between product ranks appeared in each condition and trial type.


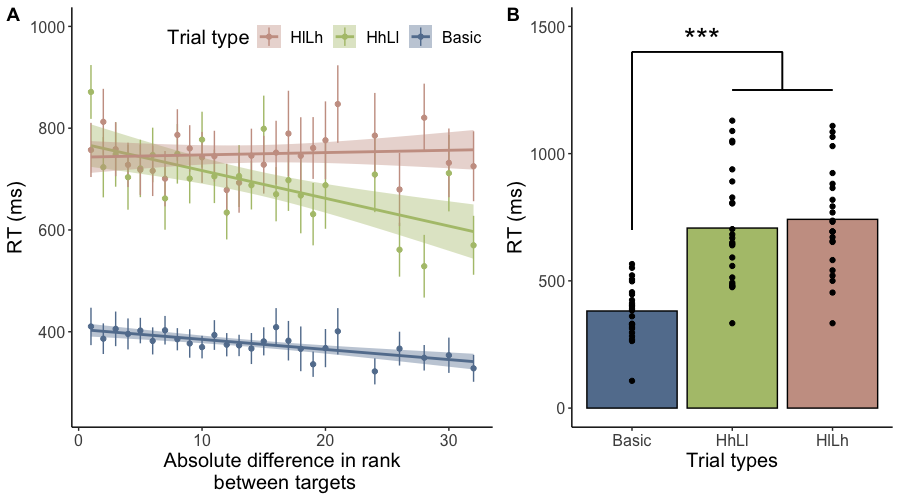


**Fig. S2.** Effect of context on RT in the Experiment 1. (A) Plots representing the RT (in ms) as a function of the absolute difference in rank between the two target products for each main trial type (Basic, HhLl, HlLh). Each dot represents the mean RT across participants in a specific rank difference in one of the trial types. The error bars represent standard errors across participants. (B) Mean RT (in ms) for each of the main trial type (Basic, HhLl, HlLh). Each dot represents the mean RT of an individual participant. *** p < .001.


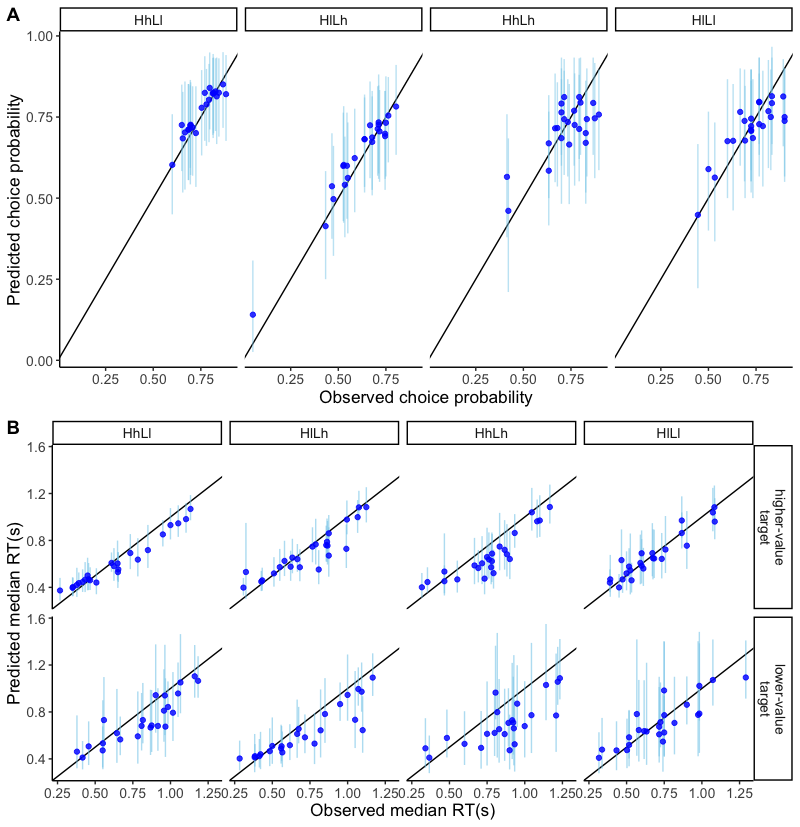


**Fig. S3.** Model fits of the best-fitting model in the Experiment 1. Plots of each participant's observed data (x-axis) versus simulated data (y-axis). We simulated the best-fitting model 500 times with the samples from the posterior distributions. We plotted (A) the probability of choosing the higher-value target and (B) the median RT of each choice. The blue dot represents the mean, and the vertical blue bar represents the 95% interval of simulated data. The black diagonal line represents the identity line (y = x). The simulated data from the best-fitting model aligns well with the observed data, showing a successful model fit.


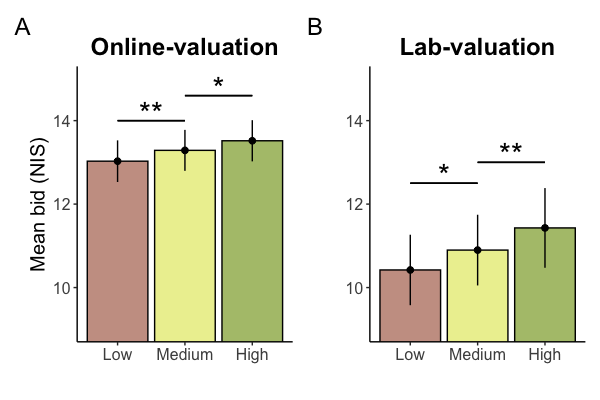


**Fig. S4.** Positive effect of context on valuation. Mean bids in each of the context trial types in (A) the *Online-valuation* experiment and in (B) the *Lab-valuation* experiment. The error bars represent standard errors across participants. * p < .05, **p < .01, *** p < .001.


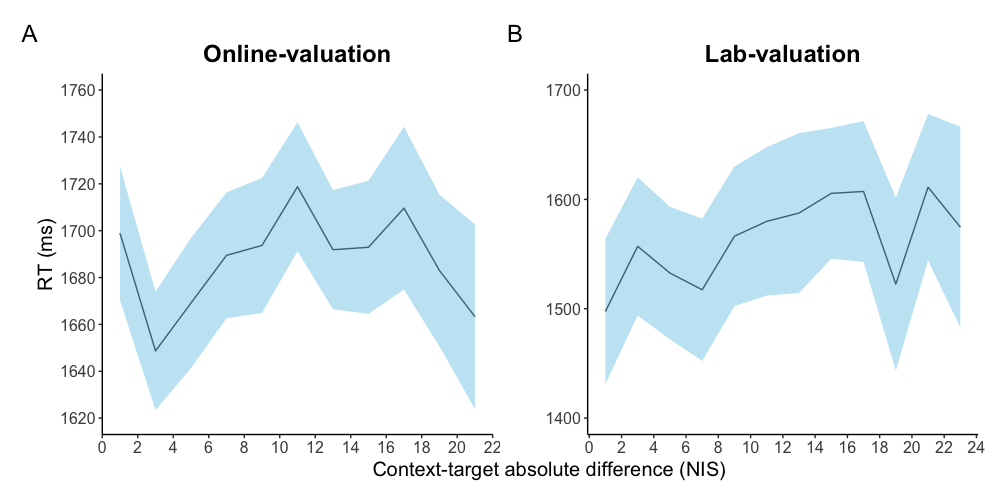


**Fig. S5.** Effect of context-target absolute difference on participants’ RT. The larger the absolute value difference between the target and the context products was, the longer it took participants to report their bid in (A) the *Online-valuation* experiment. But we did not observe a positive relationship between context-target absolute value difference and RT in (B) the *Lab-valuation* experiment. Black line represents mean RT averaged across participants. Blue shaded area represents standard errors across participants.


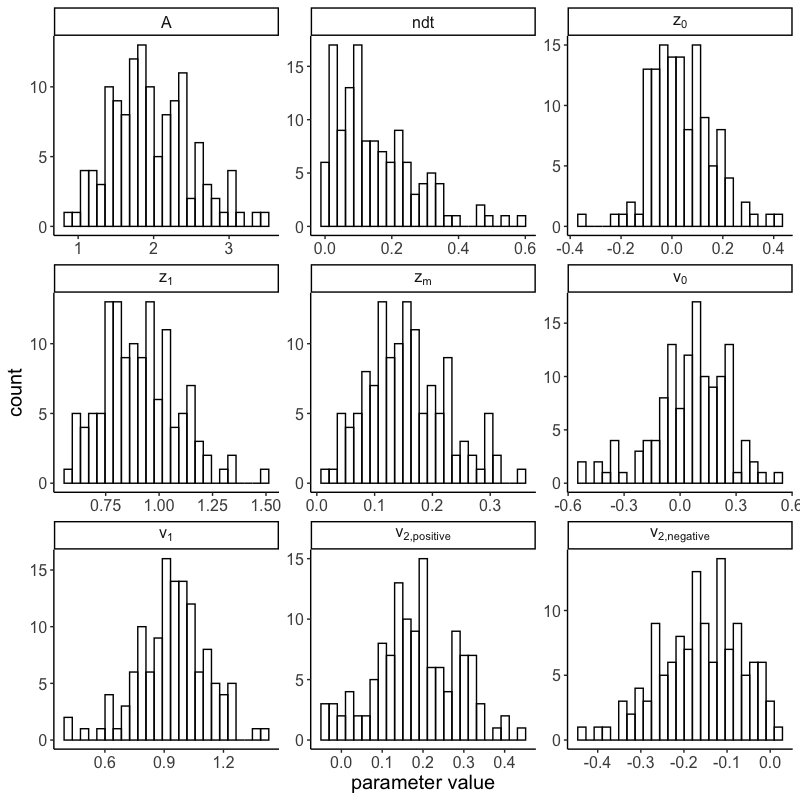


**Fig. S6.** Distribution of parameters used to simulate the WTP accumulation model.


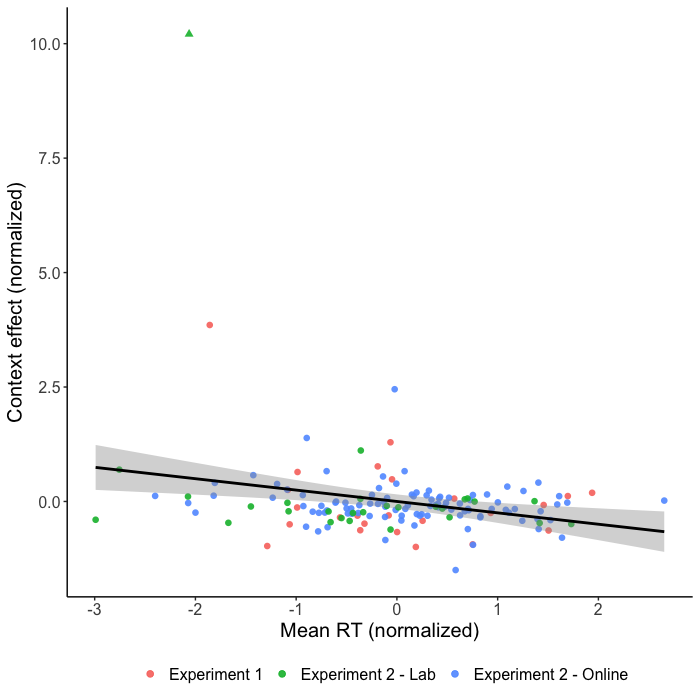


**Fig. S7.** Relationship between context effect and mean RT. Each dot represents each participant. The blue line represents a linear regression line. The grey area represents the 95% confidence interval. The green triangle is an outlier participant - excluding that participant does not change our conclusions, though it does reduce the correlation.

**Table S1.** Effect of trial type on participants’ choices in Experiment 1 (N = 24).

| **Fixed-effect Parameters** | ***B*** | **SE** | ***Z*** | **p** |
| --- | --- | --- | --- | --- |
| Intercept | 0.32 | 0.1 | 3.35 | <.001 |
| Absolute value difference | 0.14 | 0.01 | 16.73 | <.001 |
| Trial type HhLl | 0.22 | 0.08 | 2.83 | 0.005 |
| Trial type HlLh | -0.54 | 0.07 | -7.49 | <.001 |
| Trial type HhLh | -0.01 | 0.1 | -0.12 | .90 |
| Trial type HlLl | -0.01 | 0.1 | -0.11 | .91 |
| **Random-effects Parameters** | **Var** | **SE** |  |  |
| Intercept | 0.14 | 0.08 |  |  |

**Table S2.** Effect of context mean on participants’ choices in Experiment 1 (N = 24).

| **Fixed-effect Parameters** | ***B*** | **SE** | ***Z*** | **p** |
| --- | --- | --- | --- | --- |
| Intercept | 0.28 | 0.11 | 2.36 | .02 |
| Absolute value difference | 0.13 | 0.01 | 12.24 | <.001 |
| Context mean of *High* target | 0.02 | 0.003 | 5.07 | <.001 |
| Context mean of  *Low* target | -0.02 | 0.003 | -5.34 | <.001 |
| **Random-effects Parameters** | **Var** | **SE** |  |  |
| Intercept | 0.14 | 0.08 |  |  |

**Table S3.** Effect of interaction between trial type and value difference between targets on participants’ RT in Experiment 1 (N = 24).

Table S3 shows the effect of the target value difference and trial type on the RT in Experiment 1. We ran a linear regression on log-transformed RT. Regressors were the value difference between the two target products, the context trial type, and their interaction. Context trial type represents HhLl, HlLh, HhLh, and HlLl trial types relative to the Basic condition.

| **Fixed-effect Parameters** | ***B*** | **SE** | ***T*** | **p** |
| --- | --- | --- | --- | --- |
| Intercept | -0.97 | 0.05 | -19.74 | <.001 |
| Absolute value difference | -0.01 | 0.002 | -4.13 | <.001 |
| Trial type HhLl | 0.58 | 0.02 | 26.19 | <.001 |
| Trial type HlLh | 0.55 | 0.02 | 24.92 | <.001 |
| Trial type HhLh | 0.64 | 0.03 | 22.26 | <.001 |
| Trial type HlLl | 0.55 | 0.03 | 18.9 | <.001 |
| Trial type HhLl : Absolute value difference | -0.01 | 0.003 | -2.33 | .02 |
| Trial type HlLh : Absolute value difference | 0.01 | 0.003 | 3.53 | <.001 |
| Trial type HhLh : Absolute value difference | 0.01 | 0.004 | 1.61 | .11 |
| Trial type HlLl : Absolute value difference | 0.004 | 0.004 | 0.88 | .38 |
| **Random-effects Parameters** | **Var** | **SE** |  |  |
| Intercept | 0.05 | 0.02 |  |  |

**Table S4.** Choice-RT relationship in Experiment 1 (N = 24).

The table shows paired t-test results comparing the choice probabilities between HhLl and HlLh trial types in each RT bin.

| **RT bins** | **T** | **p** |
| --- | --- | --- |
| 1 | 5.30 | < .001 |
| 2 | 3.20 | .004 |
| 3 | 1.90 | .07 |
| 4 | 1.28 | .21 |
| 5 | 0.51 | .61 |

**Table S5.** Summary of models and Bayesian Predictive Information Criterion (BPIC) of Experiment 1.

Six models were fitted to the choice and RT data of the four trial types in the *Context* condition. Each model represents a unique combination of context effects on the starting point and the drift rate. For example, Model1 is a model where the starting point is constrained to be 0.5 and the drift rate is determined by the value difference of two target products.

|  | **Starting point** | **Drift rate** | **BPIC** |
| --- | --- | --- | --- |
| **Model1** | Baseline | Target-only | 6160.73 |
| **Model2** | Baseline | Context | 6060.77 |
| **Model3** | Target-only | Target-only | 6168.66 |
| **Model4** | Target-only | Context | 6068.31 |
| **Model5** | Context | Target-only | 5953.13 |
| **Model6** | Context | Context | 5925.23 |

**Table S6.** Marginal effect of context on the starting point and the drift rate.

We computed the mean BPIC of models with a specific parameter to quantify the importance of the parameter in improving model fits. For example, we computed the mean of BPIC of Model 5 and 6 to evaluate the average performance of models with context effect on the starting point. Similarly, we computed the mean BPIC of Model 2, 4, and 6 to assess the average performance of models with context effect on the drift rate. Then, we computed the difference in mean BPIC compared to the simplest model (Baseline model for starting point models and Target-only model for drift models). If a parameter leads to an improvement in model fit, the mean BPIC would be lower than that of the simplest model. Thus, the more negative ΔBPIC is, the more a parameter improves model fit.

|  | **Model variants** | **Mean BPIC** | **ΔBPIC** |
| --- | --- | --- | --- |
| **Starting point** | Baseline | 6110.75 | 0 |
|  | Target-only | 6118.48 | 7.74 |
|  | Context | 5939.18 | -171.56 |
| **Drift rate** | Target-only | 6094.17 | 0 |
|  | Context | 6018.10 | -76.07 |

**Table S7.** Group level posterior distributions of the best-fitting model.

A summary of the mean, 95% highest density interval (2.5% and 97.5% of the posterior distribution), and the proportion of posterior samples above zero (i.e. the probability of posterior distribution above zero) for the DDM parameters. Here $z_{i}$ represent the terms for the starting point. $z_{0}$ is the intercept and $z_{1}$ is the coefficient of the difference in the overall value of each side. $v_{i}$ represent the terms for the drift rate. $v_{0}$is the intercept. $v_{1}$ and $v_{2}$ are the coefficients of target value difference and context value difference, respectively.

|  | Starting point | | Drift rate | | |
| --- | --- | --- | --- | --- | --- |
|  | $z_{0}$ | $z_{1}$ | $v_{0}$ | $v_{1}$ | $v_{2}$ |
| Mean | -0.023 | 0.033 | 0.234 | 2.544 | 0.049 |
| 2.5% | -0.073 | 0.013 | 0.112 | 1.698 | -0.052 |
| 97.5% | 0.027 | 0.052 | 0.362 | 3.432 | 0.147 |
| The probability of posterior distribution above zero | 0.19 | 1 | 1 | 1 | 0.85 |

**Table S8.** Summary of individual level posterior distributions of the best-fitting model.

The table summarizes the individual level posterior distributions of the best-fitting model. The table shows the number of participants with strictly positive or negative HDI, or including zero in HDI. Also, the probability of posterior distribution above zero was averaged across participants.

|  |  | Starting point | | Drift rate | | |
| --- | --- | --- | --- | --- | --- | --- |
|  |  | $z_{0}$ | $z_{1}$ | $v_{0}$ | $v_{1}$ | $v_{2}$ |
| HDI | Strictly positive | 0 | 10 | 10 | 20 | 3 |
|  | Include zero | 24 | 14 | 14 | 4 | 20 |
|  | Strictly negative | 0 | 0 | 0 | 0 | 1 |
| The probability of posterior distribution above zero | Mean | 0.30 | 0.76 | 0.87 | 0.94 | 0.57 |
|  | Standard deviation | 0.11 | 0.27 | 0.19 | 0.18 | 0.35 |

**Table S9.** Effect of *Context-BDM* trial types on participants’ WTP in Experiment 2.

|  | **Online-valuation (N = 101)** | | | | **Lab-valuation (N = 28)** | | | |
| --- | --- | --- | --- | --- | --- | --- | --- | --- |
| **Fixed-effect Parameters** | ***B*** | **SE** | **T** | **p** | ***B*** | **SE** | **T** | **p** |
| Intercept | 0.66 | 0.22 | 3.04 | .003 | 2.06 | 0.46 | 4.44 | <.001 |
| Original bid | 0.93 | 0.01 | 101.95 | <.001 | 0.83 | 0.02 | 42.81 | <.001 |
| Low context  trial type | -0.3 | 0.1 | -3.07 | .002 | -0.48 | 0.19 | -2.45 | .01 |
| High context  trial type | 0.2 | 0.1 | 2.02 | .04 | 0.53 | 0.19 | 2.74 | .006 |
| **Random-effects Parameters** | **Var** | **SE** |  | | **Var** | **SE** |  | |
| Intercept | 2.71 | 0.41 |  | | 4.32 | 1.22 |  | |

**Table S10.** Effect of context value mean on participants’ WTP in Experiment 2.

|  | **Online-valuation (N = 101)** | | | | **Lab-valuation (N = 28)** | | | |
| --- | --- | --- | --- | --- | --- | --- | --- | --- |
| **Fixed-effect Parameters** | ***B*** | **SE** | **T** | **p** | ***B*** | **SE** | **T** | **p** |
| Intercept | 0.32 | 0.22 | 1.46 | .15 | 1.6 | 0.46 | 3.48 | .001 |
| Original bid | 0.93 | 0.01 | 101.84 | <.001 | 0.83 | 0.02 | 42.71 | <.001 |
| Context value mean | 0.02 | 0.004 | 5.48 | <.001 | 0.04 | 0.01 | 4.65 | <.001 |
| **Random-effects Parameters** | **Var** | **SE** |  | | **Var** | **SE** |  | |
| Intercept | 2.76 | 0.41 |  |  | 4.22 | 1.2 |  |  |

**Table S11.** Effect of context-target absolute difference on participants’ RTs in Experiment 2.

|  | **Online-valuation (N = 101)** | | | | **Lab-valuation (N = 28)** | | | |
| --- | --- | --- | --- | --- | --- | --- | --- | --- |
| **Fixed-effect Parameters** | **B** | **SE** | **T** | **p** | **B** | **SE** | **T** | **p** |
| Intercept | 1.66 | 0.02 | 67.52 | <.001 | 1.53 | 0.06 | 25.34 | <.001 |
| Context-target absolute difference | 0.002 | 0.001 | 2.81 | .005 | 0.001 | 0.001 | 1.18 | .24 |
| **Random-effects Parameters** | **Var** | **SE** |  | | **Var** | **SE** |  | |
| Intercept | 0.06 | 0.01 |  |  | 0.10 | 0.03 |  |  |

**Table S12.** WTP-RT relationship in Experiment 2.

The table shows results of paired t-tests comparing the mean bid between two main trial types (Low, High) in each RT bin.

|  | **Online-valuation (N = 101)** | | **Lab-valuation (N = 28)** | |
| --- | --- | --- | --- | --- |
| **RT bins** | **T** | **p** | **T** | **p** |
| 1 | -4.7 | <.001 | -2.38 | .02 |
| 2 | -1.63 | .11 | -1.74 | .09 |
| 3 | 1.05 | .30 | -1.45 | .16 |
| 4 | 0.08 | .94 | -0.98 | .33 |
| 5 | -0.87 | .39 | 0.55 | .59 |

**Table S13.** Effect of context value mean on participants’ WTP (Simulation results of the WTP accumulation model).

|  | **No context effect** | | | | **Starting point bias** | | | |
| --- | --- | --- | --- | --- | --- | --- | --- | --- |
| **Fixed-effect Parameters** | **B** | **SE** | **T** | **p** | **B** | **SE** | **T** | **p** |
| Intercept | 10.73 | 0.27 | 39.07 | <.001 | 9.86 | 0.29 | 34.02 | <.001 |
| Original bid | 0.3 | 0.01 | 20.9 | <.001 | 0.33 | 0.01 | 23.28 | <.001 |
| Context value mean | -0.003 | 0.01 | -0.46 | .65 | 0.02 | 0.01 | 3.43 | <.001 |
| **Random-effects Parameters** | **Var** | **SE** |  | | **Var** | **SE** |  | |
| Intercept | 3.84 | 0.55 |  |  | 4.89 | 0.68 |  |  |
|  | **Positive drift** | | | | **Negative drift** | | | |
| **Fixed-effect Parameters** | **B** | **SE** | **T** | **p** | **B** | **SE** | **T** | **p** |
| Intercept | 11.01 | 0.26 | 42.69 | <.001 | 10.48 | 0.28 | 37.11 | <.001 |
| Original bid | 0.27 | 0.01 | 19.52 | <.001 | 0.34 | 0.01 | 23.95 | <.001 |
| Context value mean | 0.06 | 0.01 | 9.29 | <.001 | -0.08 | 0.01 | -11.63 | <.001 |
| **Random-effects Parameters** | **Var** | **SE** |  | | **Var** | **SE** |  | |
| Intercept | 2.91 | 0.43 |  |  | 4.4 | 0.62 |  |  |
|  | **Starting point + Positive drift** | | | | **Starting point + Negative drift** | | | |
| **Fixed-effect Parameters** | **B** | **SE** | **T** | **p** | **B** | **SE** | **T** | **p** |
| Intercept | 9.72 | 0.29 | 33.34 | <.001 | 9.75 | 0.28 | 34.81 | <.001 |
| Original bid | 0.32 | 0.01 | 22.32 | <.001 | 0.32 | 0.01 | 22.65 | <.001 |
| Context value mean | 0.10 | 0.01 | 15.3 | <.001 | -0.03 | 0.01 | -4.79 | <.001 |
| **Random-effects Parameters** | **Var** | **SE** |  | | **Var** | **SE** |  | |
| Intercept | 4.96 | 0.69 |  |  | 4.19 | 0.59 |  |  |

**Table S14.** Effect of context-target absolute difference on RT (Simulation results of the WTP accumulation model).

|  | **No context effect** | | | | **Starting point bias** | | | |
| --- | --- | --- | --- | --- | --- | --- | --- | --- |
| **Fixed-effect Parameters** | **B** | **SE** | **T** | **p** | **B** | **SE** | **T** | **p** |
| Intercept | 1.31 | 0.04 | 31.89 | <.001 | 1.26 | 0.04 | 31.14 | <.001 |
| Context-target absolute difference | 0.001 | 0.0009 | 1.43 | .15 | 0.0006 | 0.0009 | 0.63 | .53 |
| **Random-effects Parameters** | **Var** | **SE** |  | | **Var** | **SE** |  | |
| Intercept | 0.21 | 0.03 |  |  | 0.2 | 0.03 |  |  |
|  | **Positive drift** | | | | **Negative drift** | | | |
| **Fixed-effect Parameters** | **B** | **SE** | **T** | **p** | **B** | **SE** | **T** | **p** |
| Intercept | 1.33 | 0.04 | 31.51 | <.001 | 1.34 | 0.04 | 31.27 | <.001 |
| Context-target absolute difference | 0.0009 | 0.0009 | 0.98 | .33 | 0.0009 | 0.0009 | 0.94 | .35 |
| **Random-effects Parameters** | **Var** | **SE** |  | | **Var** | **SE** |  | |
| Intercept | 0.22 | 0.03 |  |  | 0.22 | 0.03 |  |  |
|  | **Starting point + Positive drift** | | | | **Starting point + Negative drift** | | | |
| **Fixed-effect Parameters** | **B** | **SE** | **T** | **p** | **B** | **SE** | **T** | **p** |
| Intercept | 1.27 | 0.04 | 32.0 | <.001 | 1.26 | 0.0400 | 30.89 | <.001 |
| Context-target absolute difference | -0.001 | 0.0009 | -1.36 | .18 | 0.0002 | 0.0009 | 0.2 | .84 |
| **Random-effects Parameters** | **Var** | **SE** |  | | **Var** | **SE** |  | |
| Intercept | 0.19 | 0.02 |  |  | 0.2 | 0.03 |  |  |

**Table S15.** Relationship between mean bids and RT (Simulation results of the WTP accumulation model).

To examine the relationship between the bids and RT, we compared the mean bids for High and Low trial types in five RT bins with paired t-tests.

|  | **No context effect** | | **Starting point** | | **Positive drift** | |
| --- | --- | --- | --- | --- | --- | --- |
| **RT bins** | **T** | **p** | **T** | **p** | **T** | **p** |
| 1 | 0.01 | .99 | -6.69 | <.001 | -4.92 | <.001 |
| 2 | -0.45 | .65 | -3.53 | <.001 | -5.41 | <.001 |
| 3 | 1.02 | .31 | 0.18 | .85 | -2.35 | .02 |
| 4 | -0.49 | .62 | -0.17 | .86 | -3.22 | .002 |
| 5 | -1.08 | .28 | 1.1 | .27 | -4.76 | <.001 |
|  | **Negative drift** | | **Starting point + Positive drift** | | **Starting point + Negative drift** | |
| **RT bins** | **T** | **p** | **T** | **p** | **T** | **p** |
| 1 | 2.65 | .009 | -9.01 | <.001 | -2.98 | .003 |
| 2 | 4.36 | <.001 | -6.65 | <.001 | 0.86 | .39 |
| 3 | 3.29 | .001 | -6.61 | <.001 | 2.74 | .007 |
| 4 | 4.37 | <.001 | -6.16 | <.001 | 2.22 | .03 |
| 5 | 6.7 | <.001 | -4.86 | <.001 | 4.61 | <.001 |

**Table S16.** Experiment 2 model comparison examining lagged effects (Model with the original WTP).

The table reports model fits for regressions that predict the current trial’s WTP based on the target item’s original WTP, the average WTP for the context items, as well as the lagged versions of these variables (i.e., earlier trials’ target WTP and context WTP) and trial number. The top half of the table reports the results for the online experiment, while the bottom half reports the results for the lab experiment. Within an experiment, as we move down the table, we add an additional lagged trial. These models use the original WTP for the lagged targets. The degrees of freedom and p-value refer to the change in model complexity and improvement in fit from the current row to the row immediately above.

|  | **Model** | | **Number of parameters** | **Log likelihood** | $\boldsymbol{\chi}^{\boldsymbol{2}}$ | **Degrees of freedom** | **p-value** |
| --- | --- | --- | --- | --- | --- | --- | --- |
| **Online**  **valuation** | **Baseline** | | 5 | -38355.6 | *-* | *-* | *-* |
|  | **Sequential effect** | **t-1 only** | 8 | -38327.4 | 56.30 | 3 | <.001 |
|  |  | **up to t-2** | 10 | -38324.9 | 5.14 | 2 | .08 |
|  |  | **up to t-3** | 12 | -38324.8 | 0.11 | 2 | .95 |
|  |  | **up to t-4** | 14 | -38323.8 | 2.05 | 2 | .36 |
| **Lab**  **valuation** | **Baseline** | | 5 | -10974 | - | - | - |
|  | **Sequential effect** | **t-1 only** | 8 | -10962.3 | 23.47 | 3 | <.001 |
|  |  | **up to t-2** | 10 | -10962.2 | 0.08 | 2 | .96 |
|  |  | **up to t-3** | 12 | -10961.8 | 0.92 | 2 | .63 |
|  |  | **up to t-4** | 14 | -10961.4 | 0.76 | 2 | .68 |

**Table S17.** Experiment 2 model comparison examining lagged effects (Model with the actual response in the previous trial in the Context BDM).

The same as Table S16 except that the WTP for the lagged targets is based on the Context BDM rather than the original BDM.

|  | **Model** | | **Number of parameters** | **Log likelihood** | $\boldsymbol{\chi}^{\boldsymbol{2}}$ | **Degrees of freedom** | **p-value** |
| --- | --- | --- | --- | --- | --- | --- | --- |
| **Online**  **valuation** | **Baseline** | | 5 | -38355.6 | - | - | - |
|  | **Sequential effect** | **t-1 only** | 8 | -38319.6 | 71.91 | 3 | <.001 |
|  |  | **up to t-2** | 10 | -38317.6 | 4.07 | 2 | .13 |
|  |  | **up to t-3** | 12 | -38317.5 | 0.27 | 2 | .87 |
|  |  | **up to t-4** | 14 | -38315.2 | 4.48 | 2 | .11 |
| **Lab**  **valuation** | **Baseline** | | 5 | -10974 | *-* | *-* | *-* |
|  | **Sequential effect** | **t-1 only** | 8 | -10959.7 | 28.58 | 3 | <.001 |
|  |  | **up to t-2** | 10 | -10959.6 | 0.16 | 2 | .93 |
|  |  | **up to t-3** | 12 | -10957.2 | 4.88 | 2 | .09 |
|  |  | **up to t-4** | 14 | -10957.2 | 0.002 | 2 | 1.00 |

**Table S18.** Model coefficients for the regression in Table S16 using the “t-1” model, i.e., the model with only one trial of lagged effects.

|  | **Online-valuation (N = 101)** | | | | **Lab-valuation (N = 28)** | | | |
| --- | --- | --- | --- | --- | --- | --- | --- | --- |
| **Fixed-effect Parameters** | ***B*** | **SE** | **T** | **p** | ***B*** | **SE** | **T** | **p** |
| **Intercept** | 0.83 | 0.21 | 3.89 | <.001 | 1.99 | 0.45 | 4.47 | <.001 |
| **Original bid** | 0.91 | 0.01 | 147.58 | <.001 | 0.86 | 0.01 | 66.99 | <.001 |
| **Original bid t-1** | 0.03 | 0.01 | 4.10 | <.001 | 0.01 | 0.01 | 0.96 | .34 |
| **Context value mean** | 0.02 | 0.004 | 4.85 | <.001 | 0.04 | 0.01 | 5.00 | <.001 |
| **Context value mean t-1** | -0.01 | 0.004 | -2.19 | .03 | -0.02 | 0.01 | -2.37 | .02 |
| **Trial number** | -0.79 | 0.13 | -6.11 | <.001 | -1.09 | 0.26 | -4.17 | <.001 |
| **Random-effects Parameters** | **Var** | **SE** |  | | **Var** | **SE** |  | |
| **Intercept** | 2.31 | 0.35 |  |  | 3.56 | 1.01 |  |  |

**Table S19.** Model coefficients for the regression in Table S17 using the “t-1” model, i.e., the model with only one trial of lagged effects.

|  | **Online-valuation (N = 101)** | | | | **Lab-valuation (N = 28)** | | | |
| --- | --- | --- | --- | --- | --- | --- | --- | --- |
| **Fixed-effect Parameters** | ***B*** | **SE** | **T** | **p** | ***B*** | **SE** | **T** | **p** |
| **Intercept** | 0.76 | 0.21 | 3.71 | .001 | 1.84 | 0.44 | 4.22 | <.001 |
| **Bid** | 0.91 | 0.01 | 147.75 | <.001 | 0.86 | 0.01 | 67.08 | <.001 |
| **Bid t-1** | 0.03 | 0.01 | 5.71 | <.001 | 0.03 | 0.01 | 2.46 | .01 |
| **Context value mean** | 0.02 | 0.004 | 4.90 | <.001 | 0.04 | 0.01 | 4.97 | <.001 |
| **Context value mean t-1** | -0.01 | 0.004 | -2.44 | .02 | -0.02 | 0.01 | -2.69 | .007 |
| **Trial number** | -0.77 | 0.13 | -5.92 | <.001 | -1.05 | 0.26 | -4.03 | <.001 |
| **Random-effects Parameters** | **Var** | **SE** |  | | **Var** | **SE** |  | |
| **Intercept** | 2.16 | 0.32 |  |  | 3.34 | 0.95 |  |  |

**SI References**

1. R. Polanía, M. Woodford, C. C. Ruff, Efficient coding of subjective value. *Nat. Neurosci.* **22**, 134–142 (2019).

2. B. K. H. Chau, N. Kolling, L. T. Hunt, M. E. Walton, M. F. S. Rushworth, A neural mechanism underlying failure of optimal choice with multiple alternatives. *Nat. Neurosci.* **17**, 463–470 (2014).

3. R. Ratcliff, G. McKoon, The Diffusion Decision Model: Theory and Data for Two-Choice Decision Tasks. *Neural Comput.* **20**, 873–922 (2008).

4. T. Wiecki, I. Sofer, M. Frank, HDDM: Hierarchical Bayesian estimation of the Drift-Diffusion Model in Python. *Front. Neuroinformatics* **7** (2013).

5. P. D. Kvam, A geometric framework for modeling dynamic decisions among arbitrarily many alternatives. *J. Math. Psychol.* **91**, 14–37 (2019).

6. P. D. Kvam, J. R. Busemeyer, A distributional and dynamic theory of pricing and preference. *Psychol. Rev.* **127**, 1053–1078 (2020).

7. S. D. Brown, R. Ratcliff, P. L. Smith, Evaluating methods for approximating stochastic differential equations. *J. Math. Psychol.* **50**, 402–410 (2006).

8. F. Chen, I. Krajbich, Biased sequential sampling underlies the effects of time pressure and delay in social decision making. *Nat. Commun.* **9**, 3557 (2018).
